# Ultrafine silicon dioxide nanoparticles cause lung epithelial cells apoptosis via oxidative stress-activated PI3K/Akt-mediated mitochondria- and endoplasmic reticulum stress-dependent signaling pathways

**DOI:** 10.1038/s41598-020-66644-z

**Published:** 2020-06-18

**Authors:** Kuan-I Lee, Chin-Chuan Su, Kai-Min Fang, Chin-Ching Wu, Cheng-Tien Wu, Ya-Wen Chen

**Affiliations:** 1 Department of Emergency, Taichung Tzuchi Hospital, The Buddhist Tzuchi Medical Foundation, No.66 Section 1, Fongsing Rd., Tanzih Township, Taichung, 427 Taiwan; 2 Department of Otorhinolaryngology, Head and Neck Surgery, Changhua Christian Hospital, Changhua, 500 Taiwan; 3 School of Medicine, Kaohsiung Medical University, Kaohsiung, 807 Taiwan; 4 Department of Otolaryngology, Far Eastern Memorial Hospital, New Taipei City, 220 Taiwan; 5 Department of Public Health, China Medical University, Taichung, 404 Taiwan; 6 Department of Nutrition and Master Program of Food and Drug Safety, China Medical University, Taichung, 40402 Taiwan; 7 Department of Physiology, College of Medicine, China Medical University, No.91 Hsueh-Shih Road, Taichung, 404 Taiwan

**Keywords:** Cell death, Cell signalling, Respiration, Nanotoxicology

## Abstract

Silicon dioxide nanoparticles (SiO_2_NPs) are widely applied in industry, chemical, and cosmetics. SiO_2_NPs is known to induce pulmonary toxicity. In this study, we investigated the molecular mechanisms of SiO_2_NPs on pulmonary toxicity using a lung alveolar epithelial cell (L2) model. SiO_2_NPs, which primary particle size was 12 nm, caused the accumulation of intracellular Si, the decrease in cell viability, and the decrease in mRNAs expression of surfactant, including surfactant protein (SP)-A, SP-B, SP-C, and SP-D. SiO_2_NPs induced the L2 cell apoptosis. The increases in annexin V fluorescence, caspase-3 activity, and protein expression of cleaved-poly (ADP-ribose) polymerase (PARP), cleaved-caspase-9, and cleaved-caspase-7 were observed. The SiO_2_NPs induced caspase-3 activity was reversed by pretreatment of caspase-3 inhibitor Z-DEVD-FMK. SiO_2_NPs exposure increased reactive oxygen species (ROS) production, decreased mitochondrial transmembrane potential, and decreased protein and mRNA expression of Bcl-2 in L2 cells. SiO_2_NPs increased protein expression of cytosolic cytochrome *c* and Bax, and mRNAs expression of Bid, Bak, and Bax. SiO_2_NPs could induce the endoplasmic reticulum (ER) stress-related signals, including the increase in CHOP, XBP-1, and phospho-eIF2α protein expressions, and the decrease in pro-caspase-12 protein expression. SiO_2_NPs increased phosphoinositide 3-kinase (PI3K) activity and AKT phosphorylation. Both ROS inhibitor *N*-acetyl-l-cysteine (NAC) and PI3K inhibitor LY294002 reversed SiO_2_NPs-induced signals described above. However, the LY294002 could not inhibit SiO_2_NPs-induced ROS generation. These findings demonstrated first time that SiO_2_NPs induced L2 cell apoptosis through ROS-regulated PI3K/AKT signaling and its downstream mitochondria- and ER stress-dependent signaling pathways.

## Introduction

Silicon dioxide nanoparticles (SiO_2_NPs) was nanoform (<100 nm) of nanosilica. SiO_2_NPs are one of popular nano-materials that are broadly used in many applications, such as packaging, chemical industry, DNA and drugs delivery, cosmetics, printer toners, food additives, and cancer therapy^1–8^. It had been reported that almost 100 of consumer products containing nanosilica and nearly 1.5 million tons in the worldwide market^8–10^. However, SiO_2_NPs might induce cytotoxic effects to affect human health, especially occurs in occupational silica dust forming^11^. It has been noted that silica exposure is associated with lung fibrosis, lung cancer, emphysema, chronic obstructive pulmonary disease, or lung infections^11,12^. Inhalation was the primary route for nanosilica exposure^13^. A study has shown that SiO_2_NPs are highly toxic and can be accumulated in cytosol and endosomal compartments^14^. Other studies also discussed that nanosilica could penetrate cells, interacting with the cellular membrane and organelles^13–15^. SiO_2_NPs have been shown to induce oxidative stress and activate apoptosis in human lung epithelial derived-A549 cells^16^. Induction of oxidative stress elevates reactive oxygen species (ROS) generation that may trigger the cytotoxic pathways to cause lung epithelial cell damage^17^. Nonetheless, little was known of the role of ROS and its downstream signaling pathways in SiO_2_NPs-induced cytotoxicity of pulmonary epithelial cells. Phosphoinositide 3-kinase (PI3K) converses phosphatidylinositol 3,4-triphosphase (PIP_2_) to phosphatidylinositol 3,4,5-triphosphase (PIP_3_), which phosphorylates serine/threonine kinase AKT^18^. It has been reported that PI3K/AKT signaling is one of molecular pathways in ROS-triggered cell apoptosis^19,20^. Moreover, the mitochondria- and endoplasmic reticulum (ER) stress-regulated pathways are known to be involved in lung epithelial cell apoptosis^21^. It has been shown that SiO_2_NPs induce ROS production and lead to apoptosis in human liver cells^22^. Mitochondrial dysfunction has been demonstrated to activate caspases-related cascades^23^. Therefore, it might suggest that SiO_2_NPs induced ROS-related mitochondrial apoptosis. The induction of ER stress-related C/EBP homologous protein (CHOP) and CHOP target genes (*BIM*, *CHAC-1*, *NOXA*, and *PUMA*) by SiO_2_NPs exposure has been found in human hepatoma cells^24^. The nanoparticles of titanium dioxide, silver, and zinc oxide have also been shown to induce ER stress in cell and animal models^25–27^. It has been found that ER stress is related to oxidative stress-regulated apoptosis^28^. However, the roles of signaling pathways mentioned above in SiO_2_NPs-induced lung epithelial cell cytotoxicity still remain to be clarified.

In this study, we tried to investigate the molecular mechanisms of SiO_2_NPs-induced cytotoxicity in lung alveolar epithelial cells. We determined whether ROS, PI3K/AKT, and signals of mitochondria and ER stress were involved in SiO_2_NPs-induced cytotoxicity and the possible upstream/downstream relationship among these molecular signals.

## Results

### SiO_2_NPs induces apoptosis in L2 alveolar epithelial cells

**Figure 1:**
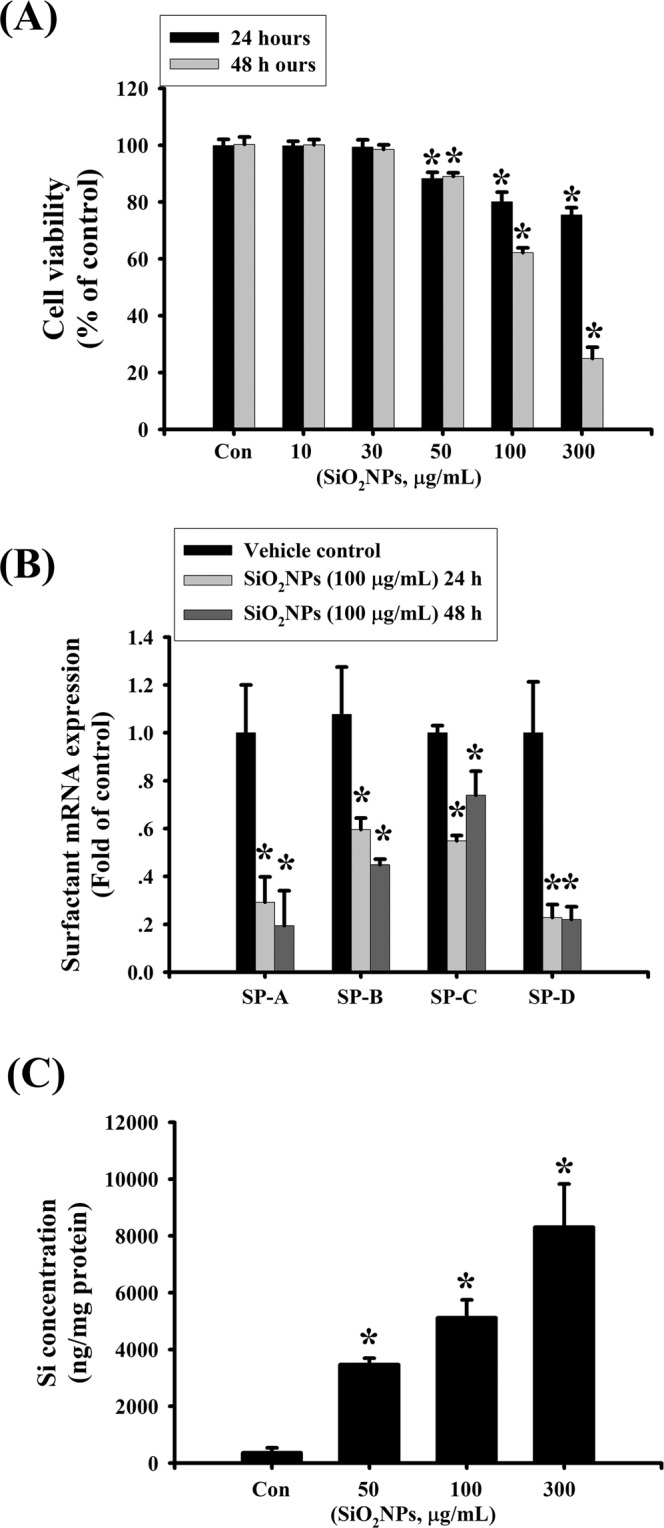
Effects of SiO_2_NPs on cells viability, surfactants mRNA expression, and intracellular Si concentration in L2 alveolar epithelial cells. (**A**) Cells were treated with SiO_2_NPs (0 to 300 μg/mL) for 24 and 48 hours. The cell viability was determined by MTT assay. (**B**) Cells were treated with SiO_2_NPs (100 μg/mL) for 24 and 48 hours. The mRNAs expression of surfactants (SPs), including SP-A, SP-B, SP-C, SP-D was determined by quantitative real-time polymerase chain reaction (qPCR) analysis. (**C**) Cells were treated with SiO_2_NPs (0 to 300 μg/mL) for 24 hours. Intracellular silicon (Si) contents were determined by inductively coupled plasma mass spectrometry (ICP-MS). All data are presented as the means ± S.D. of four independent experiments with triplicate determination. *P < 0.05 as compared to the vehicle control group. Con: control.

**Figure 2:**
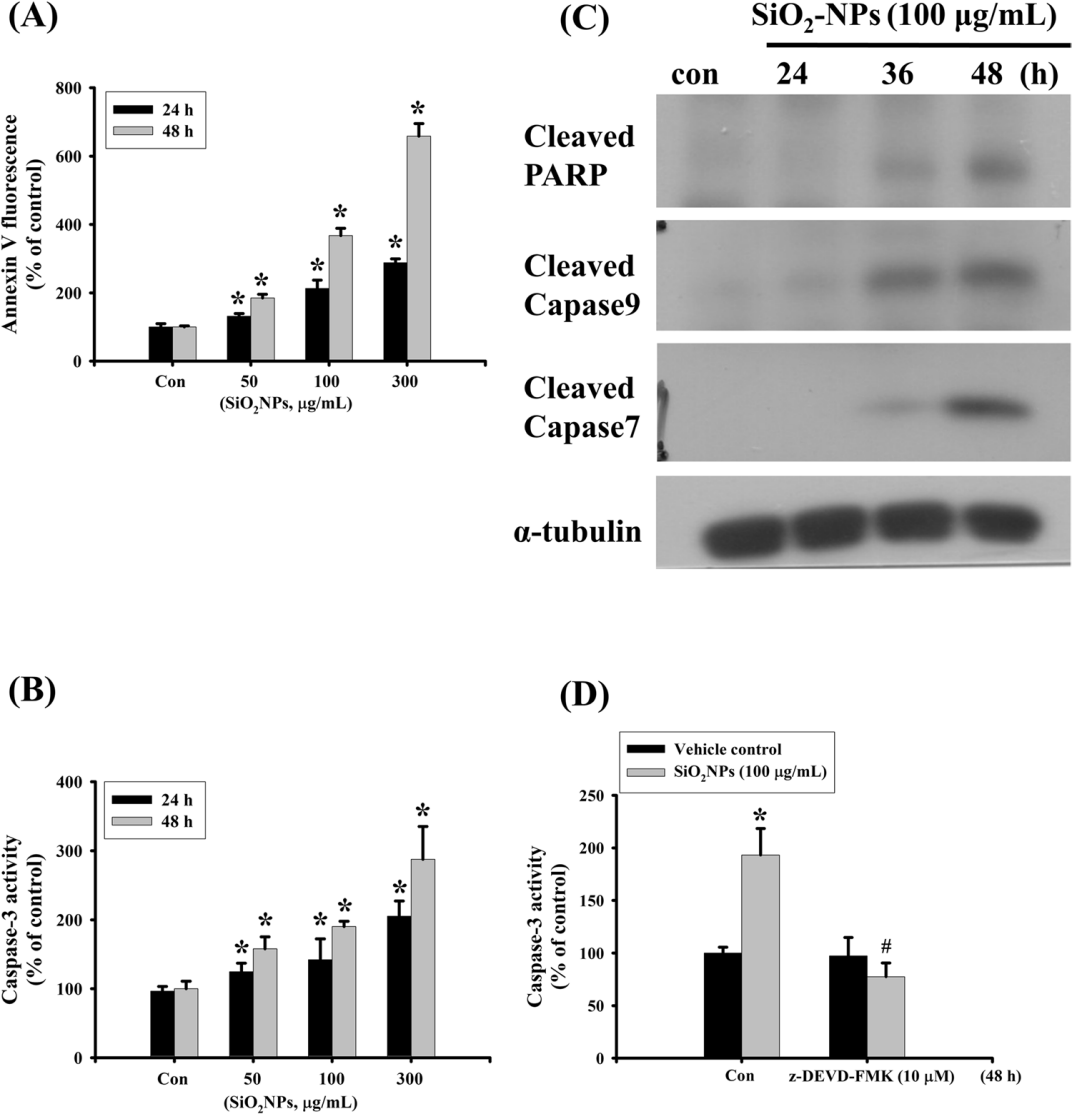
Effects of SiO_2_NPs on apoptosis signals in L2 alveolar epithelial cells. (**A**) Cells were treated with SiO_2_NPs (0 to 300 μg/mL) for 24 and 48 hours. The apoptosis was analyzed by flow cytometry with a fluorescent dye annexin V- FITC. (**B**) Cells were treated with SiO_2_NPs (0 to 300 μg/mL) for 24 and 48 hours. Caspase-3 activity was determined by Caspase-3 activity assay kit. (**C**) Cells were treated with SiO_2_NPs (100 μg/mL) for 24 to 48 hours. The protein expressions of cleaved-PARP, cleaved-caspase 9, cleaved-caspase 7 were determined by Western blotting. Data are representative of three independent experiments performed in triplicate. (**D**) Cells were pre-treated with or without Z-DEVD-FMK for 1 hour, and then treated with SiO_2_NPs for 48 hours. Caspase 3 activity was detected by Caspase-3 activity assay kit as described in the Materials and Methods. Data in (**A**,**B**,**D**) are presented as the means ± S.D. of four independent experiments with triplicate determination. *p < 0.05 as compared to vehicle control. ^#^*p* < 0.05 as compared to SiO_2_NPs groups. Con: control.

To investigate the harmful effects of SiO_2_NPs in lung cells, L2 alveolar epithelial cells were used. Cells were treated with SiO_2_NPs (10–300 μg/mL) for 24 and 48 hours. Results showed that SiO_2_NPs (100 μg/mL) significantly decreased cell viability after 24 and 48 hours treatments. Moreover, the SiO_2_NPs induced cytotoxicity in L2 alveolar cells in a dose- and time-dependent manner (Fig. 1A). We next tested the mRNA expressions of surfactants. Results showed that surfactant protein (SP)-A, SP-B, SP-C and SP-D mRNA levels were significantly reduced after 48 hours treatment of SiO_2_NPs (100 μg/mL) (Fig. 1B). We also examined the intracellular Si levels to clarify whether SiO_2_NPs could enter intracellular space. Results showed that the intracellular levels of Si in L2 cells treated with SiO_2_NPs (50–300 μg/mL) were increased in a dose-dependent manner (Fig. 1C). We next investigated the effect of SiO_2_NPs on apoptosis in L2 cells treated with SiO_2_NPs (50–300 μg/mL) for 24 and 48 hours. Results showed that SiO_2_NPs markedly increased annexin-V fluorescence (Fig. 2A) and caspase-3 activity (Fig. 2B). This increased caspase-3 activity by SiO2NPs could be reverse by caspase-3 inhibitor Z-DEVD-FMK (Fig. 2D). We also analyzed the expressions of apoptosis-related proteins in L2 cells treated with SiO_2_NPs (100 μg/mL) for 24, 36 and 48 hours. The SiO_2_NPs significantly increased cleaved-poly (ADP-ribose) polymerase (PARP), cleaved-caspase-9 and cleaved-caspase-7 protein expression (Fig. 2C). These results suggested that SiO_2_NPs was capable of inducing cytotoxicity and apoptosis in L2 cells.

### SiO_2_NPs induces ROS production and mitochondria- and ER stress-related signals in L2 alveolar epithelial cells

**Figure 3:**
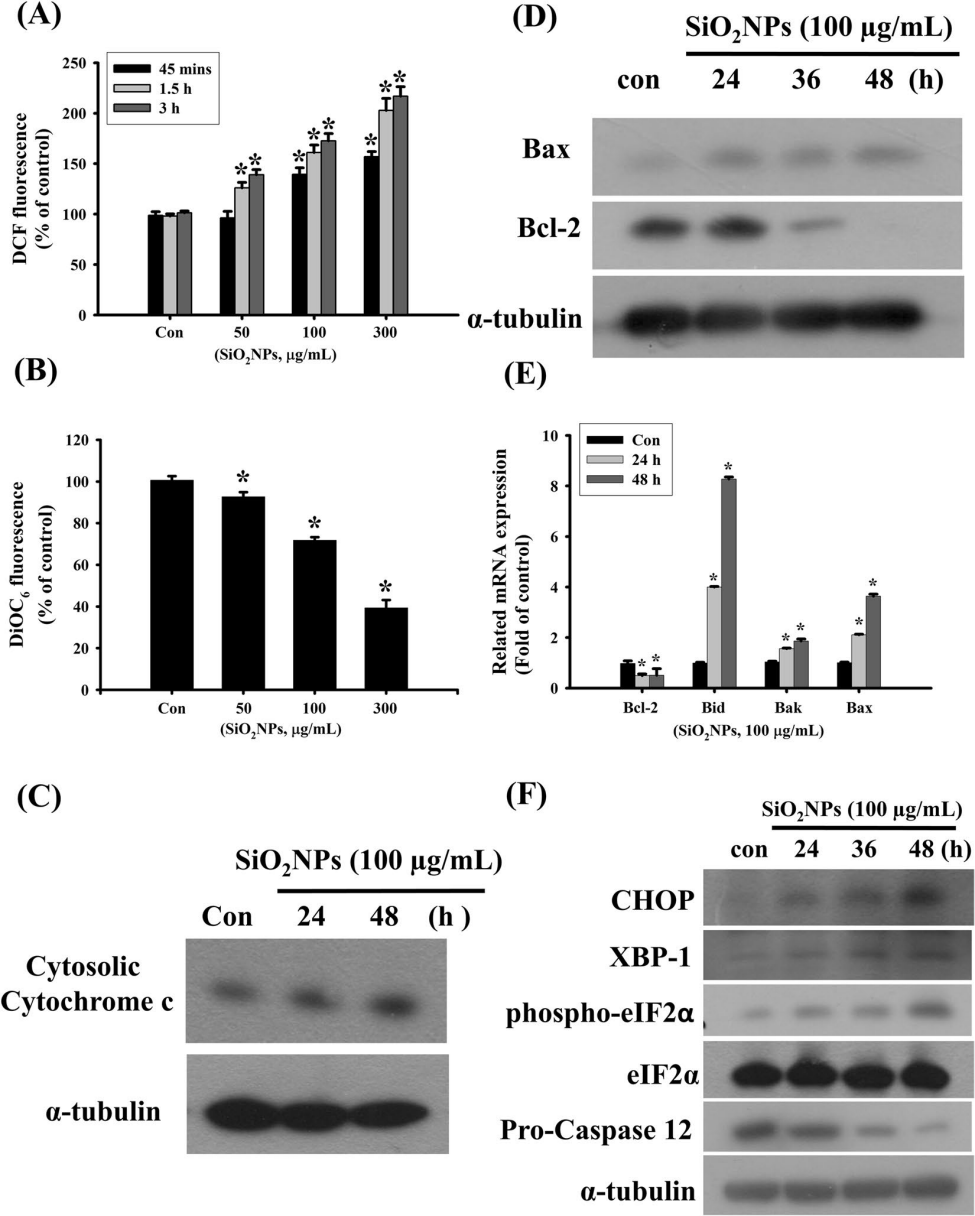
Effects of SiO_2_NPs on reactive oxygen species (ROS) production, mitochondria transmembrane potential (MMP), proteins expression of cytosolic cytochrome c, Bax, Bcl-2, and mRNAs of Bcl-2, Bid, Bak, Bax, and ER-stress related signals in L2 alveolar epithelial cells. (**A**) Cells were treated with SiO_2_NPs (100 μg/mL) for 45 minutes to 3 hours. The intracellular ROS generation was monitored by flow cytometry using peroxide-sensitive fluorescent probe (2,7 -dichlorofluorescin diacetate; DCFH-DA). (**B**) Cells were treated with SiO_2_NPs (0 to 300 μg/mL) for 24 hours. The MMP was determined by flow cytometery with a fluorescent dye DiOC_6_-FITC. Data in (**C**,**D**), cells were treated with SiO_2_NPs (100 μg/mL) for 24 to 48 hours. The proteins expressions of cytosolic cytochrome c, Bax, and Bcl-2 were determined by Western blot analysis. (**E**) Cells were treated with SiO_2_NPs (100 μg/mL) for 24 and 48 hours. The mRNA expressions of Bcl-2, Bid, Bak, and Bax were determined by quantitative real-time polymerase chain reaction (qPCR) analysis. (**F**) Cells were treated with SiO_2_NPs (100 μg/mL) for 24 to 48 hours. The proteins expressions of CHOP, XBP-1, phospho-eIF2α, pro-caspase 12 were determined by Western blot analysis. Data in (**A**,**B**,**E**) are presented as the means ± S.D. of four independent experiments with triplicate determination. *p < 0.05 as compared to vehicle control. Data in (**C**,**D**,**F**) are representative of three independent experiments performed in triplicate. Con: control.

We next investigated the potential mechanisms of SiO_2_NPs-induced cytotoxicity in L2 cells treated with SiO_2_NPs (50–300 μg/mL) for 45 minutes to 3 hours. The ROS production was analyzed by flow cytometry. Results showed that SiO_2_NPs increased ROS production in a dose- and time- dependent manner (Fig. 3A). The SiO_2_NPs (100 μg/mL) treatments also decreased mitochondrial transmembrane potential (MMP) (Fig. 3B) and increased cytosolic cytochrome c release (Fig. 3C). In the investigation of mitochondria disruptive signals, Bax protein expression was increased, and Bcl-2 protein expression was decreased in L2 cells after SiO_2_NPs (100 μg/mL) treatment (Fig. 3D). Moreover, SiO_2_NPs also caused the increase in Bid, Bak, and Bax mRNA expressions and the decrease in Bcl-s mRNA expression in L2 cells (Fig. 3E). In the investigation of ER stress-related signals, SiO_2_NPs significantly increased the protein expression of CHOP, X-box binding protein-1 (XBP-1), and phospho-eIF2α, and reduced the protein expression of pro-caspase-12 (Fig. 3F). These results suggested that SiO_2_NPs induced cell apoptosis via ROS-, mitochondria-, and ER stress-related pathways.

### The PI3K is involved in SiO_2_NPs-induced cell apoptosis

**Figure 4:**
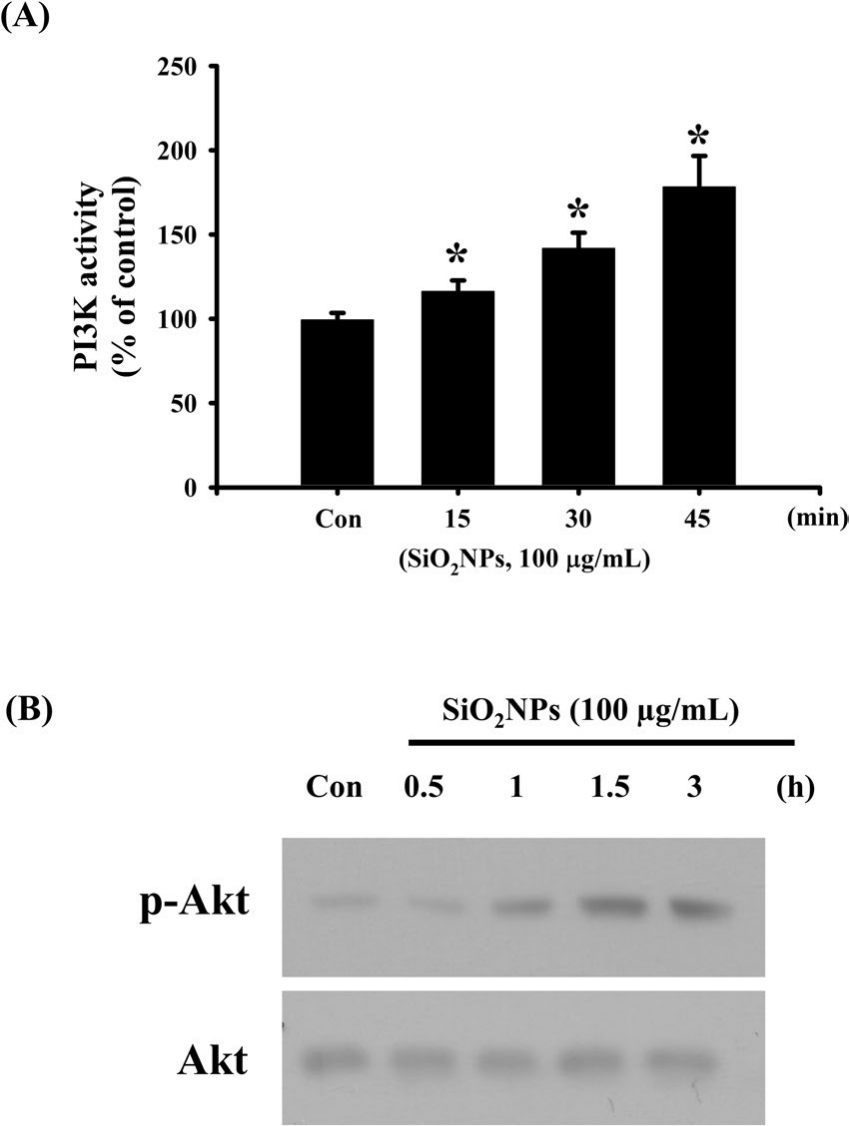
Effects of SiO_2_NPs on PI3K activity and protein expression of AKT in L2 alveolar epithelial cells. (**A**) Cells were treated with SiO_2_NPs (100 μg/mL) for 15 to 45 minutes. PI3K activity was determined by FACE PI3 Kinase Kits. Data are presented as the means ± S.D. of four independent experiments with triplicate determination. *p < 0.05 as compared to vehicle control. (**B**) Cells were treated with SiO_2_NPs (100 μg/mL) for 0.5 to 6 hours. The protein expression of phospho-AKT was determined by Western blot analysis. Data are representative of three independent experiments performed in triplicate. Con: control.

**Figure 5:**
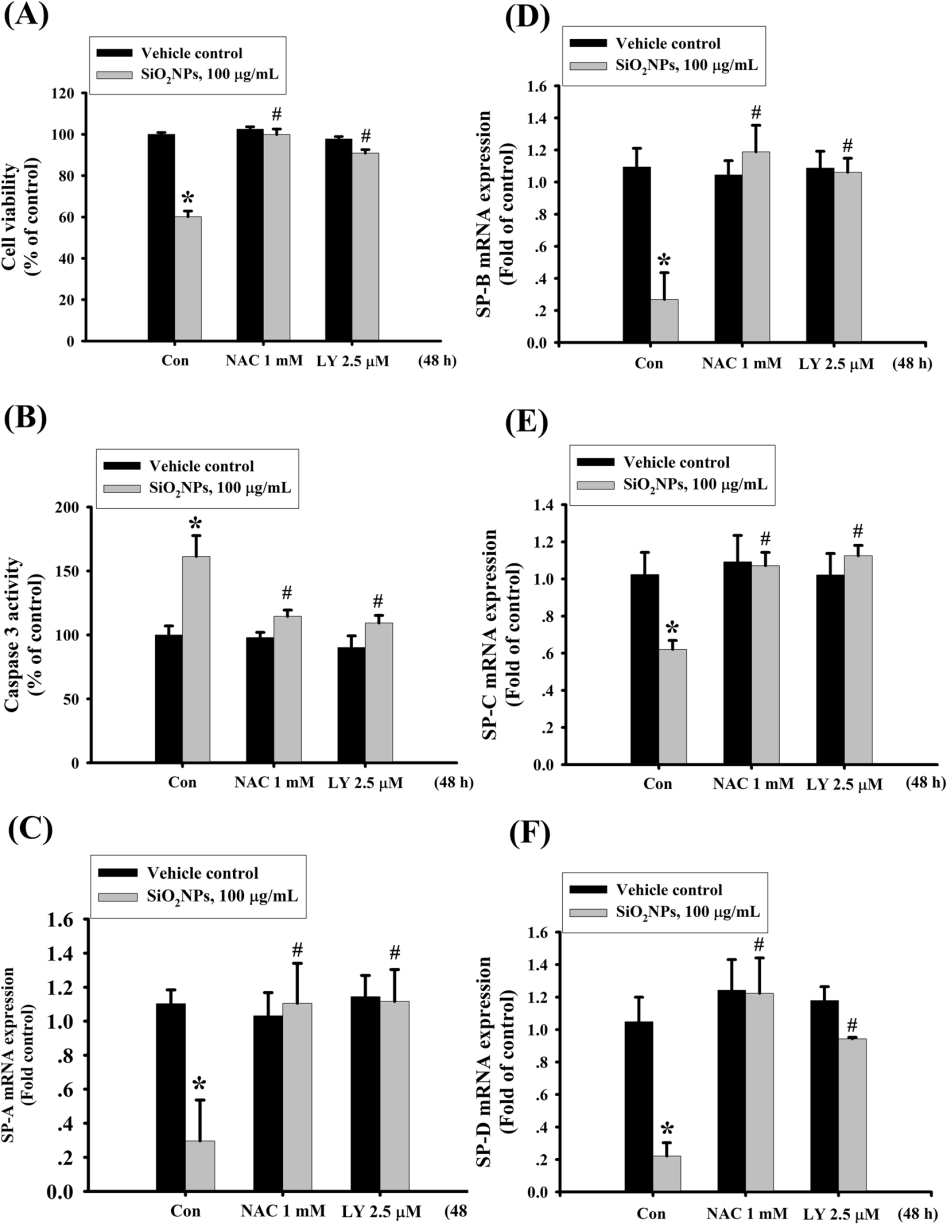
Effects of antioxidant NAC and PI3K inhibitor LY294002 on cells viability, caspase-3 activity, and surfactants (SPs) mRNA expression in SiO_2_NPs-treated L2 alveolar epithelial cells. Cells were pretreated with NAC (1 mM) or LY294002 (2.5 μM) for 1 hour, and then treated with SiO_2_NPs (100 μg/mL) for 48 hours. The cells viability was measured by MTT assay (**A**). Caspase 3 activities were detected by Caspase-3 activity assay kit (**B**). The mRNA expressions of SP-A (**C**), SP-B (**D**), SP-C (**E**), and SP-D (**F**) were measured by quantitative real-time polymerase chain reaction (qPCR) analysis. All data are presented as the means ± S.D. of four independent experiments with triplicate determination. *p < 0.05 as compared to vehicle control. ^#^*p* < 0.05 as compared to SiO_2_NPs groups.

**Figure 6:**
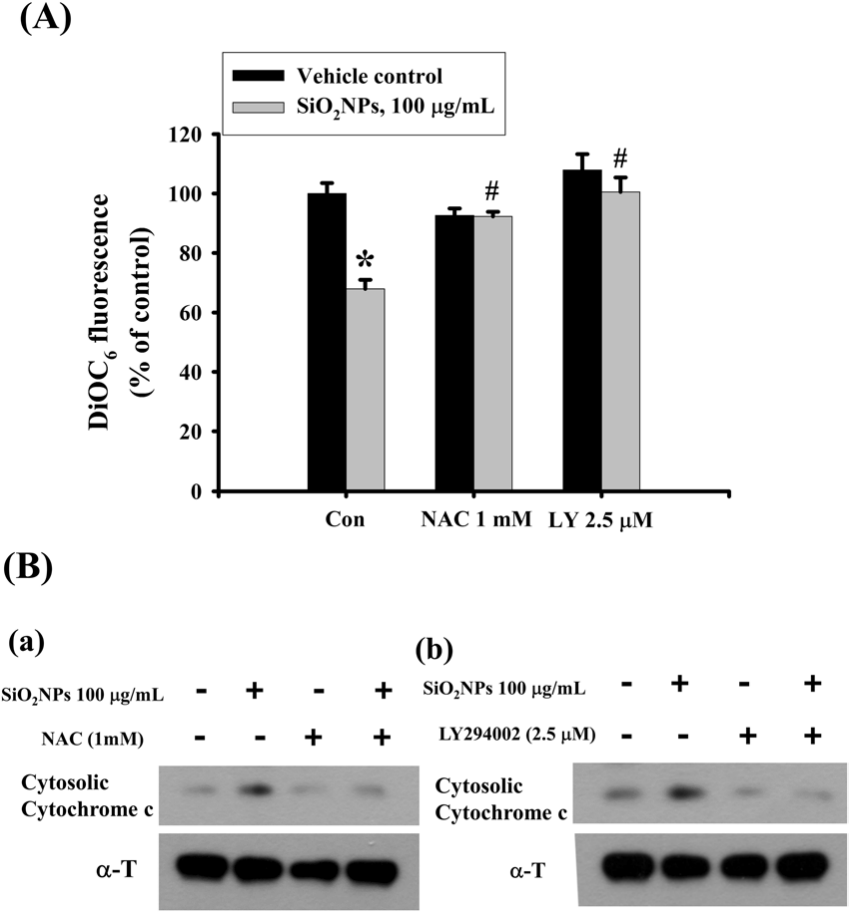
Effects of antioxidant NAC and PI3K inhibitor LY294002 on mitochondrial transmembrane potential (MMP) and cytosolic cytochrome c protein expression in SiO_2_NPs-treated L2 alveolar epithelial cells. Cells were pretreated with NAC (1 mM) or LY294002 (2.5 μM) for 1 h, and then treated with SiO_2_NPs (100 μg/mL) for 24 hours. The MMP was determined by flow cytometery with a fluorescent dye DiOC_6_-FITC (**A**). Data are presented as the means ± S.D. of four independent experiments with triplicate determination. *p < 0.05 as compared to vehicle control. ^#^*p* < 0.05 as compared to SiO_2_NPs groups. The cytosolic cytochrome c protein expression was determined by Western blot analysis (**B**-a and -b). Data are representative of three independent experiments performed in triplicate.

**Figure 7:**
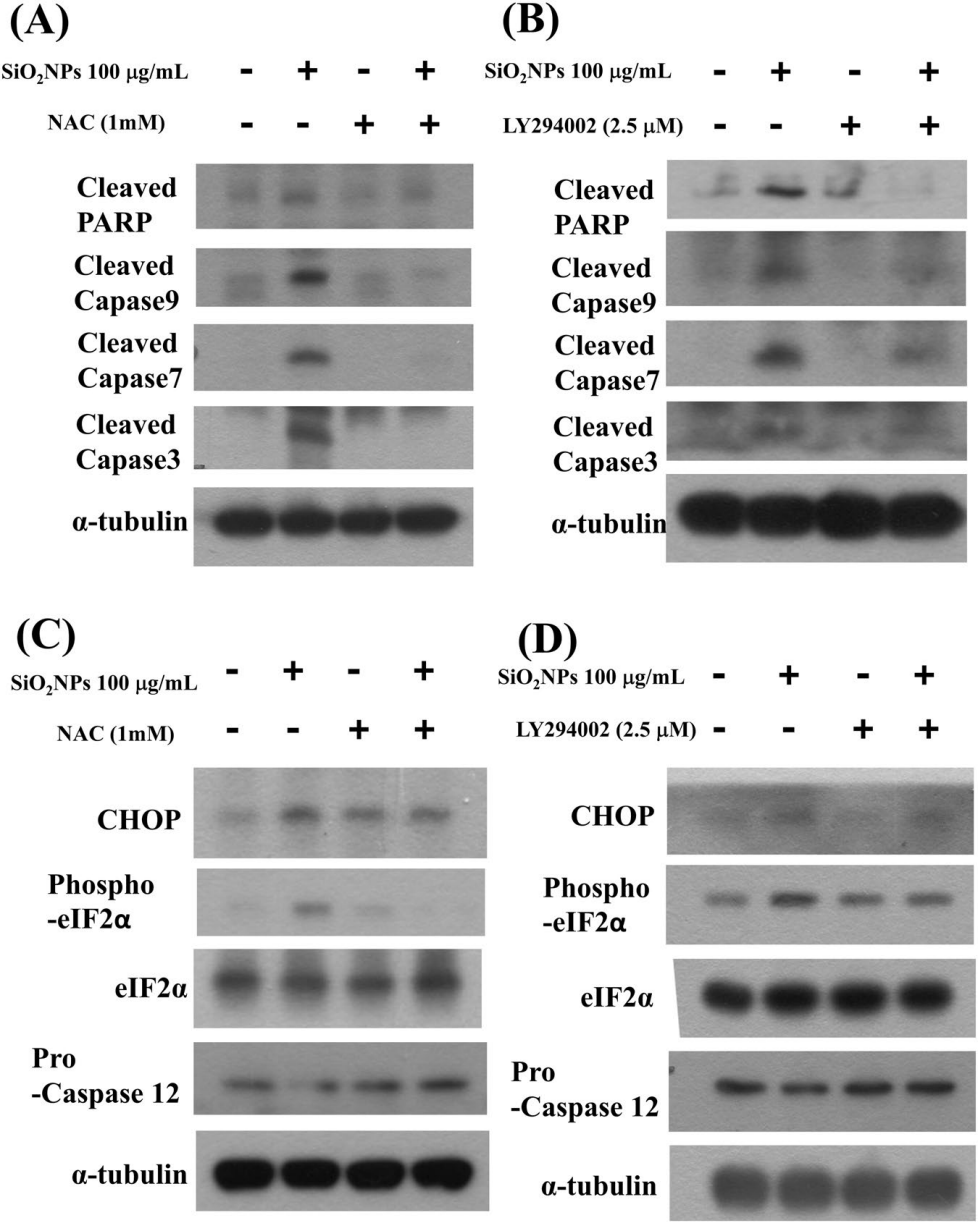
Effects of antioxidant NAC and PI3K inhibitor LY294002 on PARP, caspases signals, and ER- stress related signals in SiO_2_NPs-treated L2 alveolar epithelial cells. Cells were pretreated with NAC (1 mM) or LY294002 (2.5 μM) for 1 hour, and then treated with SiO_2_NPs (100 μg/mL) for 48 hours. The protein expression was analysis by Western blot analysis. (**A**,**B**), the protein expressions of cleaved-PARP, cleaved-caspase 9, and cleaved-caspase 7 were shown. (**C**,**D**), the protein expressions of CHOP, phospho-eIF2α, eIF2α, and pro-caspase 12 were shown. All data are representative of three independent experiments performed in triplicate.

**Figure 8:**
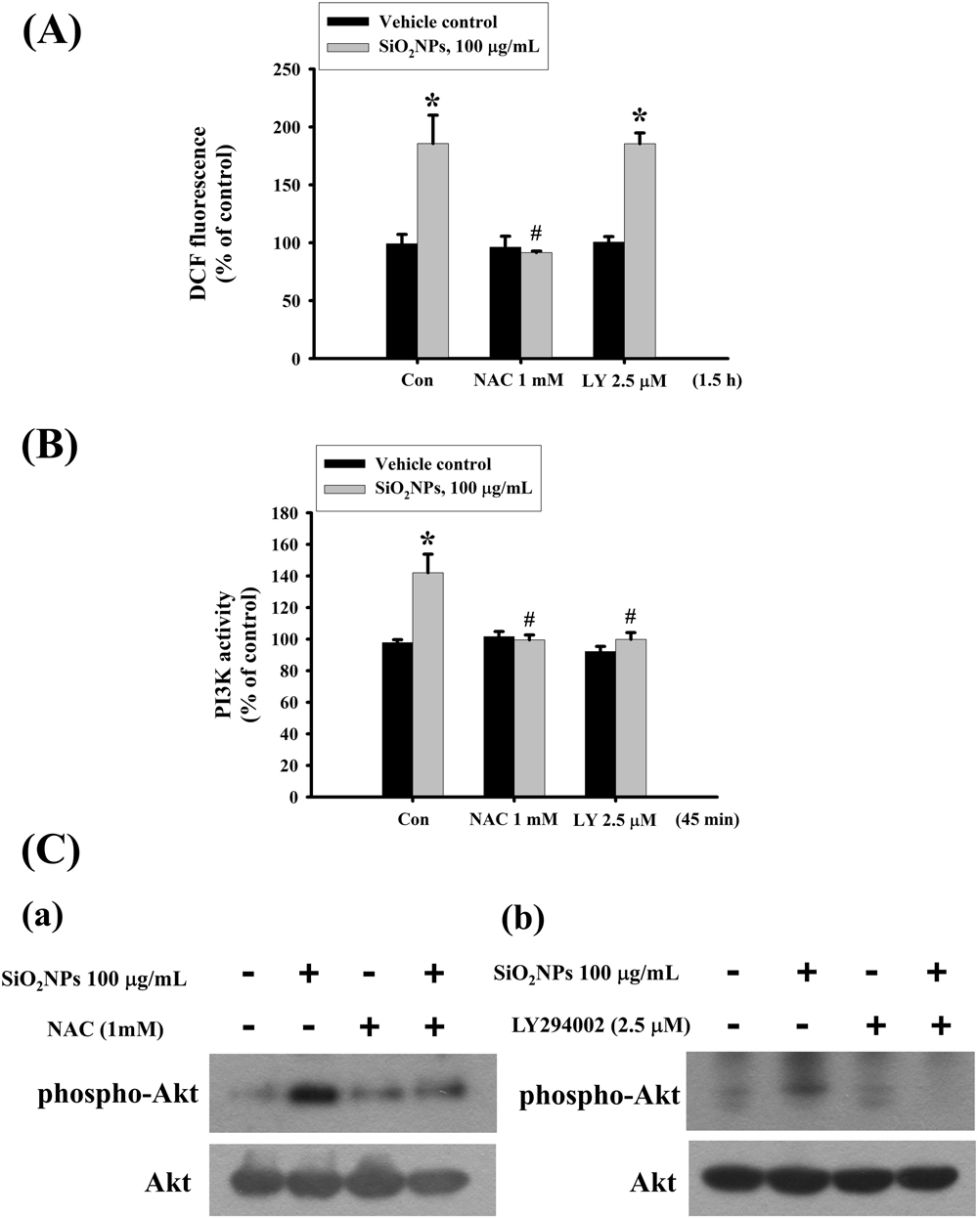
Effects of antioxidant NAC and PI3K inhibitor LY294002 on ROS production, PI3K activity, and AKT protein expression in SiO_2_NPs-treated L2 alveolar epithelial cells. Cells were pretreated with NAC (1 mM) or LY294002 (2.5 μM) for 1 h, and then treated with SiO_2_NPs (100 μg/mL). (**A**) The intracellular ROS generation was monitored by flow cytometry using peroxide-sensitive fluorescent probe (2,7 -dichlorofluorescin diacetate; DCFH-DA). (**B**) The PI3K activity was determined by FACE PI3 Kinase Kits. Data are presented as the means ± S.D. of four independent experiments with triplicate determination. *p < 0.05 as compared to vehicle control. ^#^*p* < 0.05 as compared to SiO_2_NPs groups. (**C**) The protein expression of phospho-AKT was determined by Western blot analysis. Data are representative of three independent experiments performed in triplicate.

We next tested the role of PI3K/AKT signaling in SiO_2_NPs-induced cytotoxicity. Results showed that PI3K activity (Fig. 4A) and AKT phosphorylation (Fig. 4B) was increased in L2 cells treated with SiO_2_NPs (100 μg/mL). In addition, both antioxidant *N*-acetyl-l-cysteine (NAC) (1 mM) and PI3K inhibitor LY294002 (2.5 μM) treatment significantly reversed the decreased cell viability (Fig. 5A), the increased caspase-3 activity (Fig. 5B), the decreased SP-A (Fig. 5C), SP-B (Fig. 5D), SP-C (Fig. 5E), and SP-D (Fig. 5F) mRNA expressions, the decreased MMP (Fig. 6A), the increased cytosolic cytochrome c release (Fig. 6B-a,-b), the increased protein expressions of cleaved-PARP, cleaved-caspase-9, cleaved-caspase-7, cleaved-caspase-3 (Fig. 7A,B), CHOP, and phospho-eIF2α, and the decreased pro-caspase-12 protein expression (Fig. 7C,D). We further found that the ROS production was inhibited by NAC pretreatment, but not by LY294002 pretreatment, in SiO_2_NPs-treated cells (Fig. 8A). However, both NAC and LY294002 pretreatment inhibited SiO_2_NPs-increased PI3K activity (Fig. 8B) and AKT phosphorylation (Fig. 8C). These results suggested that SiO_2_NPs triggered L2 cell apoptosis via ROS-activated PI3K/Akt-mediated mitochondria- and endoplasmic reticulum stress-dependent signaling pathways.

## Discussion

The nanosized paticles, which diameters was less than 100 nm, are easily exposure to human by various routes, such as inhalation (respiratory tract), ingestion (gastrointestinal tract), dermal (skin), and injection (blood circulation)^29^. SiO_2_NPs have applications in many industrial and medical areas. SiO_2_NPs have been found to cause adverse effects in human, such as lung fibrosis, chronic bronchitis, chronic obstructive pulmonary disease (COPD), and lung cancer^30–32^. In the present study, we elucidated the mechanisms of SiO_2_NPs-induced cytotoxicity in L2 alveolar cells that SiO_2_NPs-induced cell apoptosis via the ROS-activated PI3K/AKT signaling-mediated mitochondria- and ER stress-dependent signaling pathways.

The SiO_2_NPs-induced systemic toxicity is controversy. In a food additives study, rodents with oral administration of silica nanoparticles at a dose of 2500 mg/kg body weight did not cause the adverse health effects^33^. Beside, in a study of subacute inhalation toxicity test, exposure of rats with silica nanoparticles (0.407 ± 0.066 mg/m^3^ to 5.386 ± 0.729 mg/m^3^) for 28 days did not find the histological changes in lung tissues and the inflammatory responses in bronchoalveolar lavage fluid^34^. Yet, other studies demonstrated that oral exposure of silica nanoparticles induced liver injuries, including fatty liver, periportal liver fibrosis, and liver weight decrease^33,35,36^. van der Zande *et al*.^36^ have shown that no obviously toxic effects after animals feed with silica nanoparticles (size: 5–200 nm) 100 to 2500 mg/kg body weight were observed after 28 days exposure; however, some adverse health effects were observed after 84 days of exposure, including the increases in serum alanine aminotransferase level, lipid droplets, and periportal liver fibrosis. A study has also demonstrated that lung tissue is a major site for ^125^I labeled silica nanoparticles accumulation in mice after intravenous injection^37^. Treatment with pure silica nanoparticles (size: 50 and 100 nm) in human lung alveolar epithelial cells at the concentrations of 50 to 100 μg/mL has been shown to induce ROS generation, DNA fragmentation, and genotoxicity^38^. In the present study, we used SiO_2_NPs (size: 12 nm) 10 to 300 μg/mL to treat normal lung epithelial cells L2 for 24 and 48 hours. SiO_2_NPs induced cytotoxicity at the concentrations of 50–300 μg/mL in a dose-dependent manner. SiO_2_NPs (50–300 μg/mL) could also significantly increase the intracellular Si levels. These results indicated that SiO_2_NPs possessed cytotoxic effect and accumulative potential in lung cells as previous findings mentioned above.

The cell apoptosis is known to involve the extrinsic death receptor pathway and the intrinsic mitochondrial pathway. The mitochondria related apoptosis is resulted from the mitochondrial permeability transition pore opening and decrease mitochondrial transmembrane potential in inner mitochondria membrane in which cytochrome c can be released to cytosol and trigger the caspases-related apoptosis^39,40^. SiO_2_NPs has been found to induce cytotoxicity resulted from mitochondrial-related apoptosis in skin cancer A431 cells and lung cancer A549 cells^16^. Moreover, previous study has shown that ROS is involved in many phases of mitochondria-related apoptosis^41^. In the present study, we found that ROS elevation by SiO_2_NPs triggered mitochondrial damage, resulting mitochondrial transmembrane potential loss, cytochrome c release, and cleavages of PARP and caspases 3,7, and 9 in L2 cells. The antioxidant NAC effectively reversed the SiO_2_NPs-induced ROS-triggered mitochondria damage and apoptosis. These results suggest that oxidative stress-regulated mitochondria damage is an important risk factor in SiO_2_NPs-induced lung cell apoptosis.

Under ER stress conditions, ER-chaperone protein 78 kDa glucose-regulated protein (Grp78/BIP) is released from three dominant stress sensors, including inositol-requiring protein 1 (IRE1), PKR-like endoplasmic reticulum kinase (PERK), activating transcription factor-6 (ATF-6). Subsequently, the spliced form of XBP-1 is produced by IRE1 activation that triggers Grp78/BIP and CHOP expression. Activation of PERK phosphorylates eIF2α and increases ATF4 translation^42^. It had been shown that ER-stress was associated with various lung disorders, such as lung cancer, lung fibrosis, asthma, and lung injury^43^. Inhalation or intra-tracheal instillation of titanium dioxide, silver, or zinc oxide nanoparticles have been shown to induce ER stress^26,27,44^. However, the role of ER stress in SiO_2_NPs-induced cytotoxicity in lung epithelial cells remains unclear. In the present study, we found that ER stress-related proteins, including CHOP, XBP-1, eIF2α and caspase-12, can be upregulated or activated by SiO_2_NPs, indicating that ER stress-related signaling pathway might also contribute to SiO_2_NPs-induced cytotoxicity in L2 cells.

PI3K is a lipid kinase that involved in cell metabolism, proliferation, survival, and death^45^. AKT activation, which occurs downstream of PI3K, is known to increase ROS generation and accelerate ROS-induced apoptosis^19,46^. In the present study, we tested the relationship between PI3K/AKT signaling and ROS generation in SiO_2_NPs-induced lung alveolar cell damage. We found that SiO_2_NPs increased PI3K activity and AKT phosphorylation, which could be significantly reversed by antioxidant NAC and PI3K inhibitor LY294002. However, PI3K inhibitor LY294002 could not inhibit the ROS generation by SiO2NPs. Inhibition of ROS and PI3K/AKT signaling effectively protected lung alveolar cells against SiO_2_NPs-induced cytotoxicity and cell apoptosis. These results suggest that ROS-regulated PI3K/AKT signaling plays an important role in SiO_2_NPs-induced lung alveolar epithelial cell apoptosis.

In conclusion, the present study demonstrates that SiO_2_NPs is capable of inducing lung alveolar epithelial cell apoptosis. We further demonstrate that ROS-regulated PI3K/AKT-mediated mitochondria- and ER stress-dependent signaling pathways are involved in the SiO_2_NPs-induced cell apoptosis. These findings provide basic concerns of molecular mechanisms and possible therapeutic strategies in SiO_2_NPs-induced lung injury.

## Materials and Methods

### SiO_2_NPs

SiO_2_NPs were purchased from Sigma-Aldrich (Sigma-Aldrich, St. Louis, MO, USA). The characterization of SiO_2_NPs is 12 nm of primary particle size by transmission electron microscopy (TEM) and 99.8% of purity based on traced metal analysis. The SiO_2_NPs stock solution was modified from previous study^47^, and freshly suspended in ddH_2_O at a concentration of 5 mg/ml and then dispersed for 20 min by using a sonicator before used.

### Cell culture

The cell culture was performed as described previously^48^. Rat lung epithelial derived L2 cells were purchased from ATCC (CCL-149). Cells were cultured in RPMI-1640 media supplemented with 10% fetal bovine serum and 1% penicillin-streptomycin in 75 cm^2^ flask, and under a 5% CO_2_ and 95% air mixture at 37 ^o^C in a humid chamber. When growth density was reached 80%, cells were washed twice with PBS, and detached with 0.25% (w/v) trypsin-0.53 mM EDTA solution for 5 to 15 min. The aliquot of cells was added to a new flask or wells for next experiments.

### Cell viability

Cells were cultured in 24-well (2 × 10^5^ cells/well) and treated with SiO_2_-NPs for 24 and 48 hours. Subsequently, cells were washed twice in PBS and added fresh media with 30 µL of 3-(4,5-dimethyl thiazol-2-yl-)-2,5-diphenyl tetrazolium bromide (MTT; Sigma, St. Louis, MO, USA) (2 mg/mL) for 4 hours. Media were then removed and dimethyl sulfoxide was added to dissolve blue formazan crystals. The fluorescence was determined by using an enzyme linked immunosorbent assay (ELISA) reader (Bio-Rad, model 550, Hercules, CA, USA) at an absorption band of 570 nm.

### Intracellular silicon (Si) concentration analysis

To determine the Si levels in cells, cells were cultured in 10 cm^2^ dishes and treated with various dose of SiO_2_NPs for 24 hours. Subsequently, cells were harvested and placed in a 15 mL polyethylene tube with 0.5 mL of a 3:1 mixture of hydrochloric acid (35%) and nitric acid (70%), frozen at 220 °C, overnight. Tubes were thawed at 37 °C for 20 mins and centrifuged at 1000 × g at 4 °C for 10 mins. The Si content in supernatants was determined by inductively coupled plasma mass spectrometry (ICP-MS).

### RT-PCR analysis

The mRNAs expression of surfactant was analyzed by real-time quantitative RT-PCR (qPCR) as previously described^49^. Briefly, total intracellular RNA was extracted using RNeasy Mini kit (Qiagen Inc., USA), according to the instructions provided, according to the instructions provided, and was heated to 90 °C for 5 min to remove any secondary structures and then rapidly placed on ice. The samples were reverse transcribed into cDNA using the AMV RTase (reverse transcriptase enzyme, Promega Corporation, Pty. Ltd., USA) system. cDNA (2 μL) was tested with Real-time Sybr Green PCR reagent (Invitrogen, USA) with rat specific primers (as shown in Table 1). The amplification was performed using an ABI StepOnePlus sequence detection system (PE, Applied Biosystems, CA, USA). Data analysis was performed using StepOne software (Version 2.1, Applied Biosystems, CA, USA).

**Table 1:** Primer sequences used for the real-time quantitative RT-PCR analysis.

Primer name		Primer sequence	Reference
SP-A	Forward (5′ $\to$ 3′)	5′-GGAAGCCCTGGGATCCCTGGA-3′	^51^
	Reverse (5′ $\to$ 3′)	5′-TGGGTACCAGTTGGTGTAGT-3′	
SP-B	Forward (5′ $\to$ 3′)	5′-GTTCCACTGCAGATGCCATTG-3′	^51^
	Reverse (5′ $\to$ 3′)	5′-CATGTGCTGTTC CACAAACTG-3′	
SP-C	Forward (5′ $\to$ 3′)	5′-GATTACTCGACAGGTCCCAGGAGCCAGTTTCG-3′	^51^
	Reverse (5′ $\to$ 3′)	5′-TGGCTTATAGGCGGTCAGGAGCCGCTGGTA-3′	
SP-D	Forward (5′ $\to$ 3′)	5′- ACTTCCAGACAGTGCTGCTCTGAGGC-3′	^52^
	Reverse (5′ $\to$ 3′)	5′-ATAACCAGGCGCTGCTCT CCACAAGCC-3′	
Bcl-2	Forward (5′ $\to$ 3′)	5′-CTTTGTGGAACTGTACGGCCCCAGCATGCG-3′	^52^
	Reverse (5′ $\to$ 3′)	5′-ACAGCCTGCAGCTTTGTTTCATG-GTACATC-3	
Bid	Forward (5′ $\to$ 3′)	5′-CACGACCGTGAACTTTAT-3′	^52^
	Reverse (5′ $\to$ 3′)	5′-GCTGTTCTCTGGGACC-3′	
Bak	Forward (5′ $\to$ 3′)	5′-TTTGGCTACCGTCTGGCC-3′	^52^
	Reverse (5′ $\to$ 3′)	5′-GGCCCAACAGAACCACACC-3′	
Bax	Forward (5′ $\to$ 3′)	5′-GGGAATTCTGGAGCTGCAGAGGATGATT-3′	^52^
	Reverse (5′ $\to$ 3′)	5′-GCGGA TCCAAGTTGCCATCAGCAAACAT-3′	

### Caspase-3 activity analysis

Cells were cultured at a density of 2 × 10^5^ cells/well and treatment of SiO_2_NPs with or without antioxidant NAC or PI3K inhibitor LY294002 for 24 hours. Subsequently, cells were lysed and cell lysates were incubated with caspase-3/CPP32 substrate, Ac-DEVD-AMC (10 μM) (Promega Corporation, Madison, WI, USA) for 1 h, 37 °C. The fluorescence of cleaved substrate was detected by spectrofluorometer (Spectramax, Molecular Devices, CA, USA) at excitation wavelength 380 nm and emission wavelength 460 nm. The protein concentration was determined by using bicinchoninic acid (BCA) protein assay kit (Pierce, Rockford, IL, USA) to normalize the cell numbers between control and others groups.

### Flowcytometry analysis

Apoptosis, ROS production and mitochondrial transmembrane potential (MMP) in SiO_2_NPs treated cells were evaluated by flow cytometer. After cells were treated SiO_2_NPs with or without NAC or LY294002 for 24 h, cells were harvested and washed twice with PBS. Cells were stained with Annexin V-FITC (Biovision Research Products, Moutain View, CA) for 20 mins at room temperature. Subsequently, cells were washed twice with PBS and the fluorescence of apoptosis was detected by flow cytometeric analysis. To detection of ROS generation, cells were stained with 2′,7′-dicholorofluorescein diacetate (DCF-DA, Sigma, St. Louis, MO, USA) for 30 mins at 37 °C. The DCF-DA entered to cytosol and converted to hydrophilic 2,7-dichloroflurorescein (DCFH) by cytosolic esterase. The fluorescence of peroxide oxidized DCFH was detected by flow cytometeric analysis. To assess MMP alteration, cells were stained with DiOC_6_ for 30 mins at 37 °C, and analyzed by flowcytometer (Becton-Dickinson, Franklin Lakes, NJ, USA).

### PI3K activity assay

PI3K activity was executed according to manufacturer’s protocol (Active Motif). Cells were cultured in wells with approximately 80% confluent and treated with SiO_2_NPs. After, cells were washed twice of PBS and fixed with 4% formaldehyde in PBS for 20 min at room temperature, and then formaldehyde was removed and washed with wash buffer. Blocking buffer was supplemented with samples and incubated for 1 hour at room temperature. After rinsing with PBS, all samples were incubated with primary phospho-PI3K antibody at 4 °C, overnight. Subsequently, primary antibody was removed and incubated with HRP-conjugated secondary for 1 hour at room temperature. Then, the developing solution was supplemented with each well and incubated for 15 minutes at room temperature. The phospho-PI3K absorbance of 450 nm was read on a spectrophotometer.

### Western blot analysis

Western blot analysis was performed as described previously^50^. Equal amount of protein samples (50 μg) were resolved on SDS-PAGE and transferred to polyvinylidine difluoride (PVDF) membrane. The blots were blocked with PBST (PBS and 0.05% Tween 20) containing 5% nonfat dry milk for 1 hour at room temperature, and then probed with antibodies against cleaved-PARP, cleaved-caspase 9, cleaved-caspase 7, cytochrome c, Bax, Bcl-2, CHOP, XBP-1, phospho-eIF2α, pro-caspase 12, phospho-AKT, AKT, α-tubulin for 1 hour at 4 °C. After, membranes were washed with 0.1% PBST and incubated with secondary antibodies conjugated to horseradish peroxidase for 45 min. The antibody-reactive bands were revealed using enhanced chemiluminescence reagents (Amersham Biosciences, Sweden) and exposed to radiographic film (Kodak, Rochester, NY, USA).

### Statistical analysis

The data are shown as the means ± standard deviation (S.D.). One-way ANOVA was used for the analysis of multiple groups. Duncan’s post hoc test was utilized to identify group differences. *P* values less than 0.05 were regarded as significant. The statistical package SPSS 11.0 for Windows (SPSS Inc., Chicago, IL, USA) was applied for all statistical analyses.

## Supplementary information

Supplementary Information.

## References

[1] Hirsch LR (2003). Nanoshell-mediated near-infrared thermal therapy of tumors under magnetic resonance guidance. Proc Natl Acad Sci.

[2] Bharali DJ (2005). Organically modified silica nanoparticles: a nonviral vector for *in vivo* gene delivery and expression in the brain. Proc Natl Acad Sci USA.

[3] Gemeinhart RA, Luo D, Saltzman WM (2005). Cellular fate of a modular DNA delivery system mediated by silica nanoparticles. Biotechnol Prog.

[4] Venkatesan N, Yoshimitsu J, Ito Y, Shibata N, Takada K (2005). Liquid filled nanoparticles as a drug delivery tool for protein therapeutics. Biomaterials.

[5] Napierska D, Thomassen LC, Lison D, Martens JA, Hoet PH (2010). The nanosilica hazard: another variable entity. Part Fibre Toxicol.

[6] Oberdorster G (2010). Safety assessment for nanotechnology and nanomedicine: concepts of nanotoxicology. J Intern Med.

[7] Fede C (2012). The toxicity outcome of silica nanoparticles (Ludox(R)) is influenced by testing techniques and treatment modalities. Anal Bioanal Chem.

[8] Murugadoss S (2017). Toxicology of silica nanoparticles: an update. Arch Toxicol.

[9] Liljenström, C., Lazarevic, D. & Finnveden, G. Silicon-based nanomaterials in a life-cycle perspective, including a case study on self-cleaning coatings. ISBN 978-91-7501-942-0 (2013)

[10] Vance ME (2015). Nanotechnology in the real world: Redeveloping the nanomaterial consumer products inventory. Beilstein J Nanotechnol.

[11] Leung CC, Yu IT, Chen W (2012). Silicosis. Lancet.

[12] National Institute for Occupational Safety and Health (NIOSH). Health effects of occupational exposure to respirable crystalline silica. Cincinnati, OH: Department of Health and Human Services. 129 (2002).

[13] Sanchez A (2017). Silica nanoparticles inhibit the cation channel TRPV4 in airway epithelial cells. Part Fibre Toxicol.

[14] Al-Rawi M, Diabate S, Weiss C (2011). Uptake and intracellular localization of submicron and nano-sized SiO(2) particles in HeLa cells. Arch Toxicol.

[15] Kettiger H, Schipanski A, Wick P, Huwyler J (2013). Engineered nanomaterial uptake and tissue distribution: from cell to organism. Int J Nanomedicine.

[16] Ahamed M (2013). Silica nanoparticles-induced cytotoxicity, oxidative stress and apoptosis in cultured A431 and A549 cells. Hum Exp Toxicol.

[17] Li W (2018). Polysaccharide FMP-1 from Morchella esculenta attenuates cellular oxidative damage in human alveolar epithelial A549 cells through PI3K/AKT/Nrf2/HO-1 pathway. Int J Biol Macromol.

[18] Chetram MA (2013). ROS-mediated activation of AKT induces apoptosis via pVHL in prostate cancer cells. Mol Cell Biochem.

[19] Nogueira V (2008). Akt determines replicative senescence and oxidative or oncogenic premature senescence and sensitizes cells to oxidative apoptosis. Cancer Cell.

[20] Packer L, Fuehr K (1977). Low oxygen concentration extends the lifespan of cultured human diploid cells. Nature.

[21] Chen YW, Yang YT, Hung DZ, Su CC, Chen KL (2012). Paraquat induces lung alveolar epithelial cell apoptosis via Nrf-2-regulated mitochondrial dysfunction and ER stress. Arch Toxicol.

[22] Ahmad J (2012). Apoptosis induction by silica nanoparticles mediated through reactive oxygen species in human liver cell line HepG2. Toxicol Appl Pharmacol.

[23] Ahamed M, Akhtar MJ, Khan MAM, Alhadlaq HA, Aldalbahi A (2017). Nanocubes of indium oxide induce cytotoxicity and apoptosis through oxidative stress in human lung epithelial cells. Colloids Surf B Biointerfaces.

[24] Christen V, Fent K (2016). Silica nanoparticles induce endoplasmic reticulum stress response and activate mitogen activated kinase (MAPK) signalling. Toxicol Rep.

[25] Huo L (2015). Silver nanoparticles activate endoplasmic reticulum stress signaling pathway in cell and mouse models: The role in toxicity evaluation. Biomaterials.

[26] Yang X (2015). Endoplasmic reticulum stress and oxidative stress are involved in ZnO nanoparticle-induced hepatotoxicity. Toxicol Lett.

[27] Yu KN (2015). Inhalation of titanium dioxide induces endoplasmic reticulum stress-mediated autophagy and inflammation in mice. Food Chem Toxicol.

[28] Pallepati P, Averill-Bates DA (2011). Activation of ER stress and apoptosis by hydrogen peroxide in HeLa cells: protective role of mild heat preconditioning at 40 degrees C. Biochim Biophys Acta.

[29] Oberdorster G, Oberdorster E, Oberdorster J (2005). Nanotoxicology: an emerging discipline evolving from studies of ultrafine particles. Environ Health Perspect.

[30] Arts JH, Muijser H, Duistermaat E, Junker K, Kuper CF (2007). Five-day inhalation toxicity study of three types of synthetic amorphous silicas in Wistar rats and post-exposure evaluations for up to 3 months. Food Chem Toxicol.

[31] McLaughlin JK, Chow WH, Levy LS (1997). Amorphous silica: a review of health effects from inhalation exposure with particular reference to cancer. J Toxicol Environ Health.

[32] Merget R (2002). Health hazards due to the inhalation of amorphous silica. Arch Toxicol.

[33] Winkler HC, Suter M, Naegeli H (2016). Critical review of the safety assessment of nano-structured silica additives in food. J Nanobiotechnology.

[34] Shin JH (2018). Subacute inhalation toxicity study of synthetic amorphous silica nanoparticles in Sprague-Dawley rats. Inhal Toxicol.

[35] So SJ, Jang IS, Han CS (2008). Effect of micro/nano silica particle feeding for mice. J Nanosci Nanotechnol.

[36] van der Zande M (2014). Sub-chronic toxicity study in rats orally exposed to nanostructured silica. Part Fibre Toxicol.

[37] Xie G, Sun J, Zhong G, Shi L, Zhang D (2010). Biodistribution and toxicity of intravenously administered silica nanoparticles in mice. Arch Toxicol.

[38] Bhattacharya K, Naha PC, Naydenova I, Mintova S, Byrne HJ (2012). Reactive oxygen species mediated DNA damage in human lung alveolar epithelial (A549) cells from exposure to non-cytotoxic MFI-type zeolite nanoparticles. Toxicol Lett.

[39] Asweto CO (2017). Cellular pathways involved in silica nanoparticles induced apoptosis: A systematic review of *in vitro* studies. Environ Toxicol Pharmacol.

[40] Saelens X (2004). Toxic proteins released from mitochondria in cell death. Oncogene.

[41] Chen YW (2006). Methylmercury induces pancreatic beta-cell apoptosis and dysfunction. Chem Res Toxicol.

[42] Iurlaro R, Muñoz-Pinedo C (2016). Cell death induced by endoplasmic reticulum stress. FEBS J.

[43] Marciniak SJ (2017). Endoplasmic reticulum stress in lung disease. Eur Respir Rev.

[44] Huo L (2015). Silver nanoparticles activate endoplasmic reticulum stress signaling pathway in cell and mouse models: The role in toxicity evaluation. Biomaterials.

[45] Dumont AG, Dumont SN, Trent JC (2012). The favorable impact of PIK3CA mutations on survival: an analysis of 2587 patients with breast cancer. Chin J Cancer.

[46] Tothova Z (2007). FoxOs are critical mediators of hematopoietic stem cell resistance to physiologic oxidative stress. Cell.

[47] Akhtar MJ (2010). Nanotoxicity of pure silica mediated through oxidant generation rather than glutathione depletion in human lung epithelial cells. Toxicology.

[48] Liu SH, Su CC, Lee KI, Chen YW (2016). Effects of Bisphenol A Metabolite 4-Methyl-2,4-bis(4-hydroxyphenyl)pent-1-ene on Lung Function and Type 2 Pulmonary Alveolar Epithelial Cell Growth. Sci Rep.

[49] Chung YP (2019). Methylmercury exposure induces ROS/Akt inactivation-triggered endoplasmic reticulum stress-regulated neuronal cell apoptosis. Toxicology.

[50] Chen YW (2010). Pyrrolidine dithiocarbamate (PDTC)/Cu complex induces lung epithelial cell apoptosis through mitochondria and ER-stress pathways. Toxicol Lett.

[51] Chen CM, Wang LF, Yeh TF (2005). Effects of maternal nicotine exposure on lung surfactant system in rats. Pediatr Pulmonol.

[52] Bozec A (2004). The mitochondrial-dependent pathway is chronically affected in testicular germ cell death in adult rats exposed in utero to anti-androgens. J Endocrinol.

