# Supplementary material for: Ultrafine silicon dioxide nanoparticles cause lung epithelial cells apoptosis via oxidative stress-activated PI3K/Akt-mediated mitochondria- and endoplasmic reticulum stress-dependent signaling pathways
Source: Sci Rep. 2020 Jun 18;10:9928. doi: 10.1038/s41598-020-66644-z (PMC7303152; doi:10.1038/s41598-020-66644-z)

Supplementary materials and methods, Supplementary Figure Legends and Figures, and the corresponding full-length originals of western blot analysis for ''Ultrafine silicon dioxide nanoparticles cause lung epithelial cells apoptosis via oxidative stress-activated PI3K/Akt-mediated mitochondria- and endoplasmic reticulum stress-dependent signaling pathways''

Kuan-I Lee[1,*], Chin-Chuan Su[2,3,*], Kai-Min Fang[4,*], Chin-Ching Wu[5], Cheng-Tien Wu[6], Ya-Wen Chen[7]

[1]Department of Emergency, Taichung Tzuchi Hospital, The Buddhist Tzuchi Medical Foundation, No.66 Section 1, Fongsing Rd., Tanzih Township, Taichung 427, Taiwan.

[2]Department of Otorhinolaryngology, Head and Neck Surgery, Changhua Christian Hospital, Changhua 500, Taiwan.

[3]School of Medicine, Kaohsiung Medical University, Kaohsiung 807, Taiwan.

[4]Department of Otolaryngology, Far Eastern Memorial Hospital, New Taipei City 220, Taiwan.

[5]Department of Public Health, China Medical University, Taichung 404, Taiwan.

[6]Department of Nutrition and Master Program of Food and Drug Safety, China Medical University, Taichung 40402, Taiwan

[7]Department of Physiology, College of Medicine, China Medical University, No.91 Hsueh-Shih Road, Taichung 404, Taiwan.

*These authors contributed equally to this study.

Address correspondence to Ya-Wen Chen, Department of Physiology, College of Medicine, China Medical University, Taichung, Taiwan. E-mail:d91447001@ntu.edu.tw; Tel.: +886 4 22053366 ext. 2223.

Supplementary materials and methods

Animals. To establish the SiO_2NPs exposured mouse model, four-week-old C57BL/6 male mice was obtained from BioLASCO Taiwan Co., Ltd (Taipei, Taiwan). All protocols used were approved by the Institutional Animal Care and Use Committee (IACUC), and the care and use of laboratory animals conducted in accordance with the guidelines of the Animal Research Committee of China Medical University, Taiwan. Mice was housed in a room at a constant temperature of 22 ± 2°C with a 12 h light-dark cycle. The mice were randomly assigned to pretreatment groups, weighed, and administered with the indicated drugs or vehicle. The mice were randomly distributed into four groups: (a) vehicle control, (b) SiO_2NPs 6.2 mg/kg, (c) SiO_2NPs 12.5 mg/kg, (b) SiO_2NPs 31 mg/kg. Mice placed under deep anesthetized by intraperitoneal injection of zoletil 50 (40 mg/kg). The SiO_2NPs was diluted by PBS, and 50 μL of mixture exposed to mice via intranasal route. The mortality rate of mice was observed after treated with SiO_2NPs for 1 to 8 days.

Wet -to -dry weight ratio. At day 8 of the experiment completion, all lungs were dissected free of nonpulmonary tissue and weighed and then dried to a constant weight at 60°C. Wet-to-dry (W/D) ratios will be obtained by dividing the wet weight by the final dried weight[53].

Lipid peroxidation assay. Lung tissues were harvested from mice under zoletil 50 anesthesia (40 mg/kg i.p.). All samples were homogenized and centrifuged at 3000 g for 10 min at 4°C. The cell lysate were homogenized and centrifuged at 1000 rpm for 20 min at 4°C.

Collecting the supernatant and assays were carried out immediately using the lipid peroxidation assay kit (Calbiochem). Absorbance at 586 nm for malondialdehyde(MDA) and 405 nm for MPO was measured using an ELISA microplate reader[54].

Histological evaluation. The lung tissue was graded by Animal Disease Diagnostic Center College of Veterinary Medicine, National Chung Hsing University, Taiwan. The alteration of edema, hemorrhage, leukocyte infiltrate and necrosis in lung tissue was observed by hematoxylin and eosin (H&E) staining. Histological changes will be scored by counting the frequency of foci per field observed at 40X, using a 0-to 4-point scale with injury in 0, 25, 50, 75 or 100% of the investigated tissue. The scoring scale is as follows: 0 (absent), 1 (mild), 2 (moderate), 3 (severe) and 4 (overwhelming).

Figure legend of Supplementary Data

Supplementary Figure 1. Effects of SiO_2NPs on cells viability and caspase-3 activity in A549 human alveolar epithelial cells. (A) Cells were treated with SiO_2NPs (0 to 1000 μg/mL) for 24 hours. The cell viability was determined by MTT assay. (B) Cells were pretreated with NAC (1 mM) or LY294002 (2.5 μM) for 1 hour, and then treated with SiO_2NPs (400 μg/mL) for 48 hours. Caspase 3 activity was detected by Caspase 3 activity assay kit as described in the Materials and Methods. All data are presented as the means ± S.D. of four independent experiments with triplicate determination. *$p < 0.05$ as compared to vehicle control. #$p < 0.05$ as compared to SiO_2NPs groups. Con: control.

Supplementary Figure 2. Effects of SiO_2NPs on ROS generation and protein expression of phospho-AKT, cleaved caspase-3 in A549 human alveolar epithelial cells. Cells were pretreated with NAC (1 mM) or LY294002 (2.5 μM) for 1h, and then treated with SiO_2NPs (400 μg/mL) for 1.5 hours. (A) The intracellular ROS generation was monitored by flow cytometry using peroxide-sensitive fluorescent probe (2,7 -dichlorofluorescin diacetate; DCFH-DA). (B) The protein expression of phospho-AKT was determined by Western blot analysis. Data in (A), are presented as the means ± S.D. of four independent experiments with triplicate determination. *$p < 0.05$ as compared to vehicle control. Data in (B), are representative of three independent experiments performed in triplicate.

Supplementary Figure 3. The mortality rate of mice after SiO_2NPs treatment. C57BL/6 male mice were instilled via intranasal route with PBS, and 6.2 mg/kg, 12.5 mg/kg, 31 mg/kg of SiO_2NPs. (A) The mortality rate was determined after SiO_2NPs treatment for 1 to 8 days. (B) The wet -to -dry weight ratio was determined after SiO_2NPs treatment at day 8. (C) The alteration of MDA levels were determined after SiO_2NPs treatment at day 8. All data are presented as the means ± S.D.; n = 16 for all groups. *$p < 0.05$ as compared to vehicle control. Con: control.

Supplementary Figure 4. Histopathology of lung tissues (H&E staining) after SiO_2NPs treatment at day 8 in mice. C57BL/6 male mice were instilled via intranasal route with PBS, and 6.2 mg/kg, 12.5 mg/kg, 31 mg/kg of SiO_2NPs. (A) 20x, (B) 100x, (C) 400x, show lung tissue of control, and 12.5 mg/kg, 31 mg/kg of SiO_2NPs group. Lung tissue 12.5 mg/kg, 31 mg/kg of SiO_2NPs group shows marked inflammation and focal hemorrhage in terminal bronchial (arrows).

Supplementary Figure 5. The mRNA expression of lung tissues in mice after SiO_2NPs treatment. C57BL/6 male mice were instilled via intranasal route with PBS, and 6.2 mg/kg, 12.5 mg/kg, 31 mg/kg of SiO_2NPs. The lung tissues were collected at day 8 and the mRNA

expression of caspase-3 (A), caspase-7 (B), and caspase-9 (C) was determined by quantitative real-time polymerase chain reaction (qPCR) analysis. All data are presented as the means ± S.D.; n = 16 for all groups. *$P < 0.05$ as compared to the vehicle control group. Con: control.

Supplementary Figure 6. The mRNA expression of lung tissues in mice after SiO_2NPs treatment. C57BL/6 male mice were instilled via intranasal route with PBS, and 6.2 mg/kg, 12.5 mg/kg, 31 mg/kg of SiO_2NPs. The lung tissues were collected at day 8 and the mRNA expression of CHOP (A), XBP-1 (B), Grp78 (C), Grp94 (D), and capase-12 (E) was determined by quantitative real-time polymerase chain reaction (qPCR) analysis. All data are presented as the means ± S.D.; n = 16 for all groups. *$P < 0.05$ as compared to the vehicle control group. Con: control.

Supplementary Figure 1

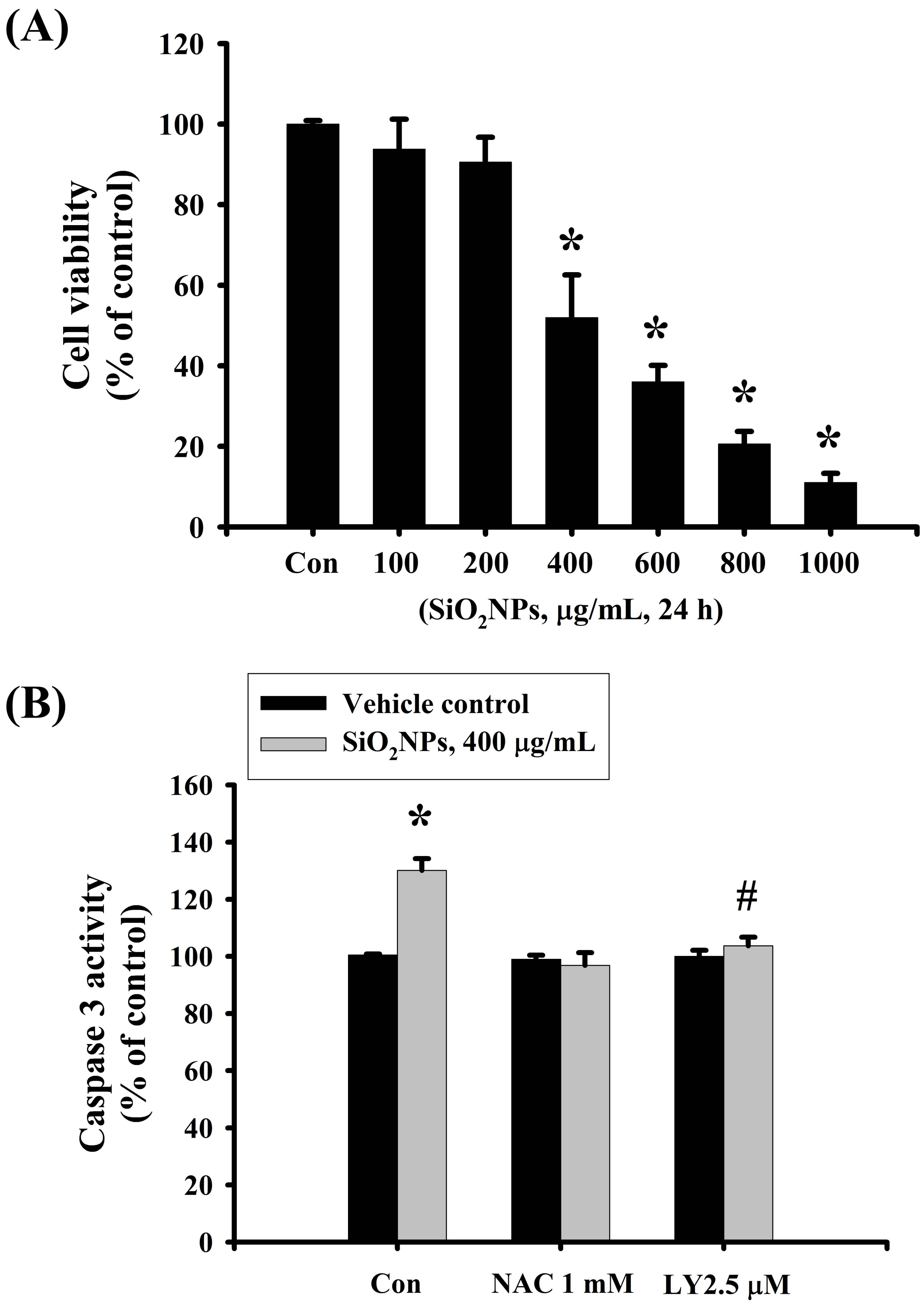

Supplementary Figure 2

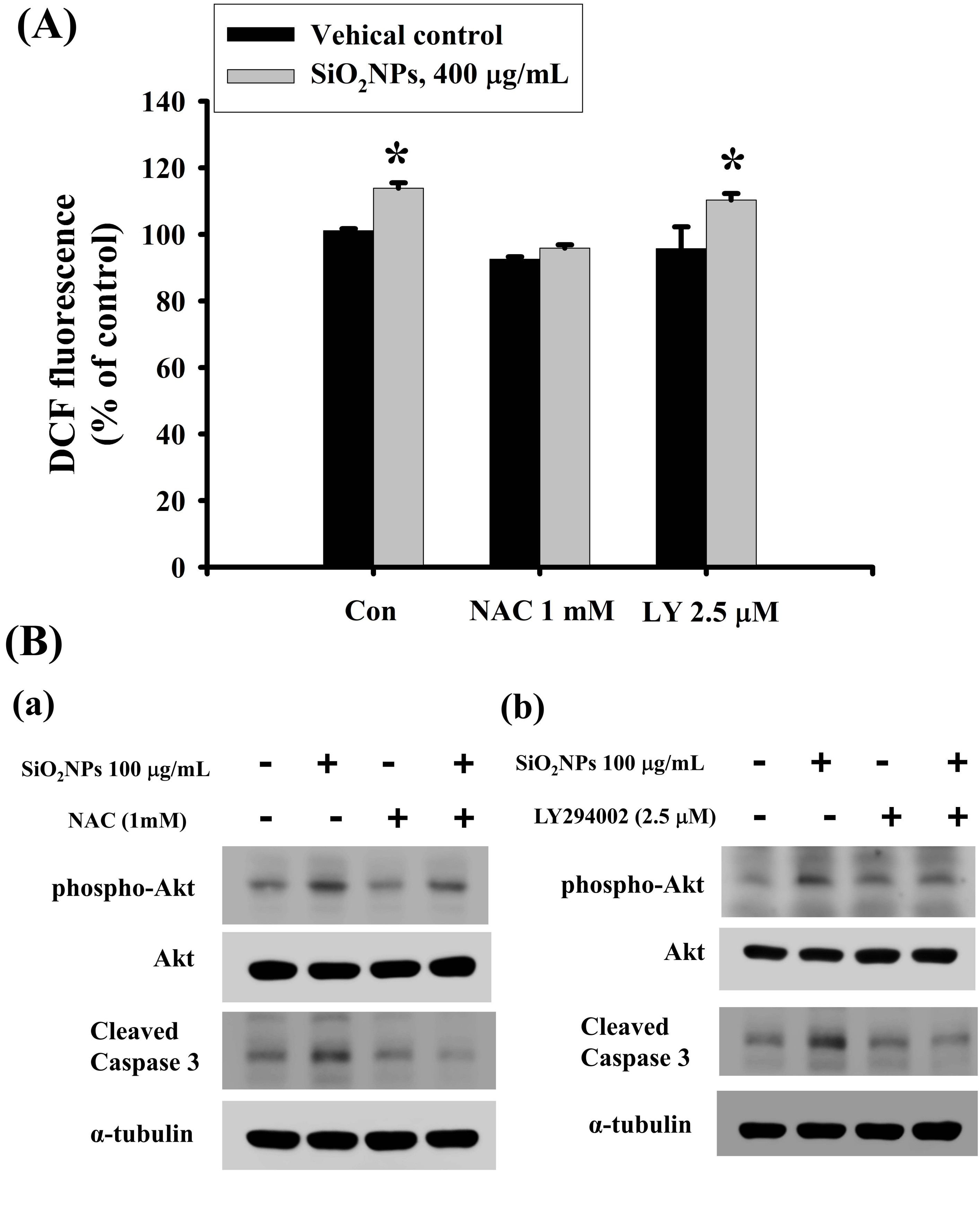

Supplementary Figure 3

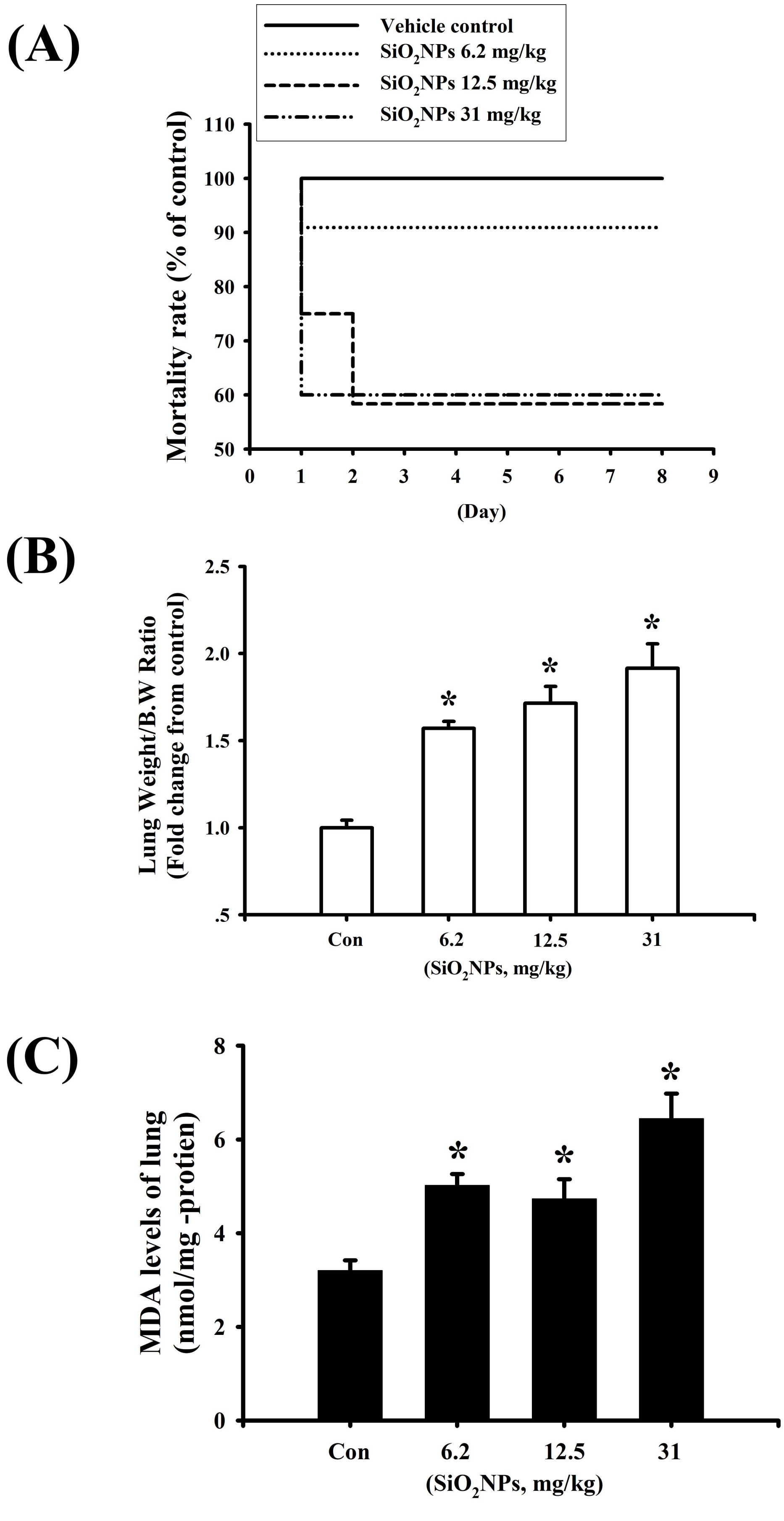

Supplementary Figure 4

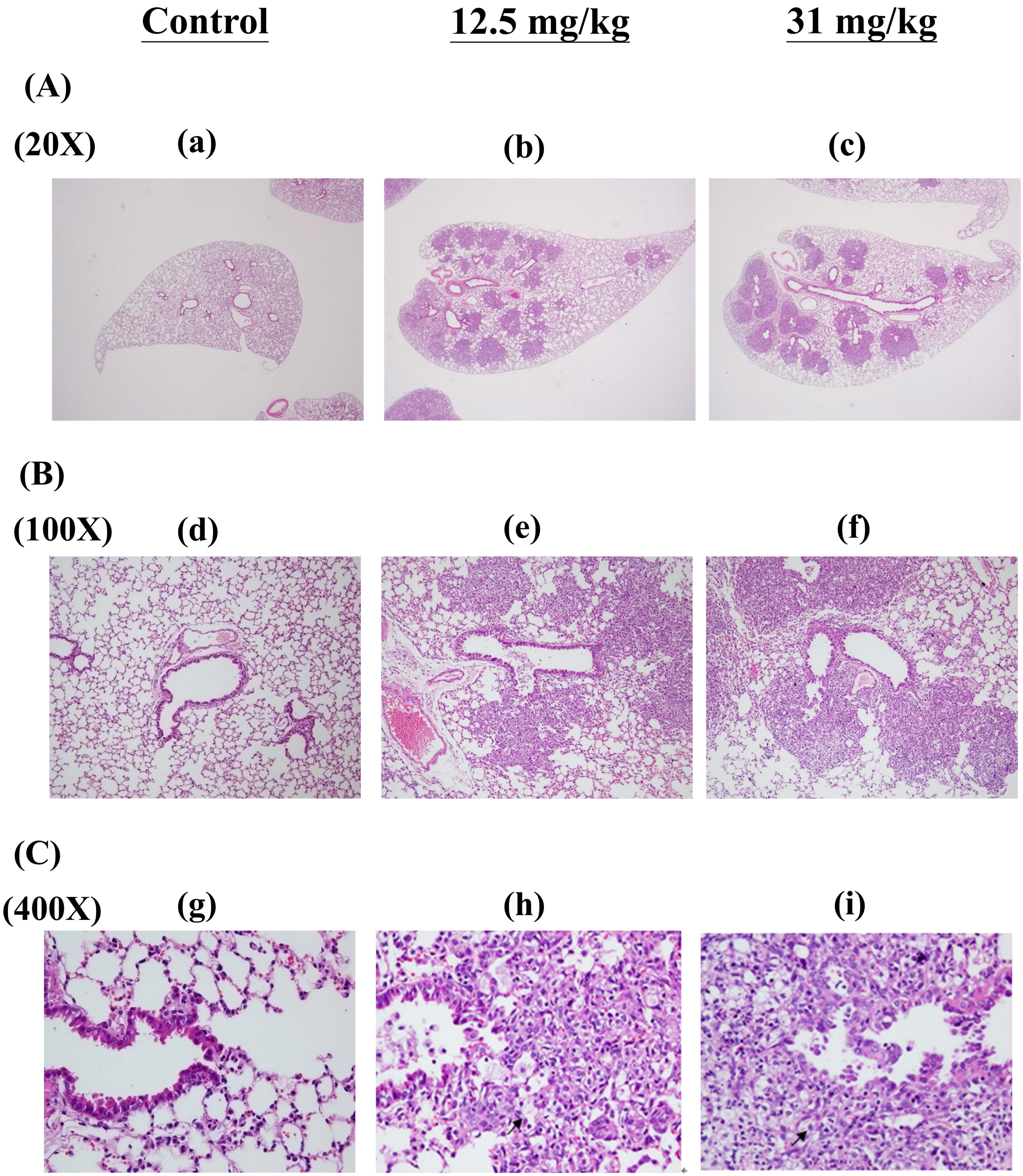

Supplementary Figure 5

(A)

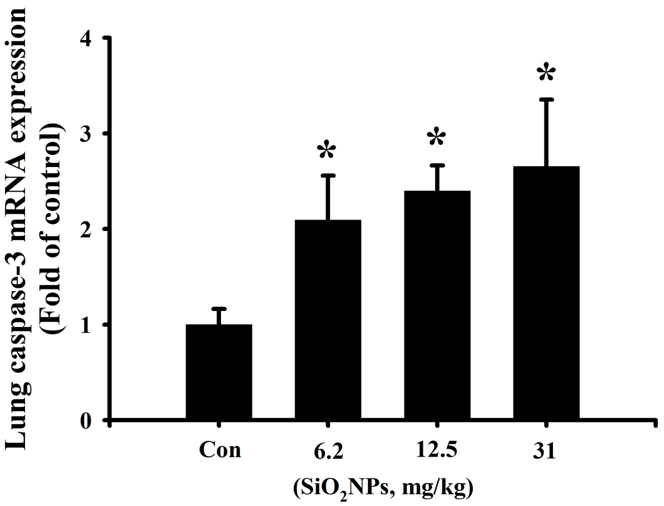

(B)

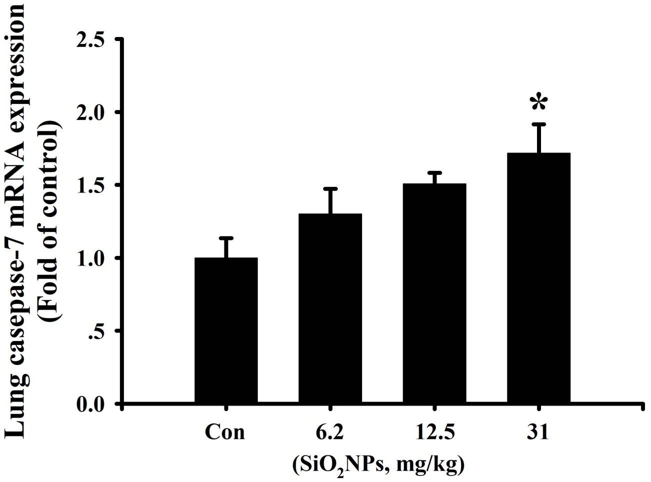

(C)

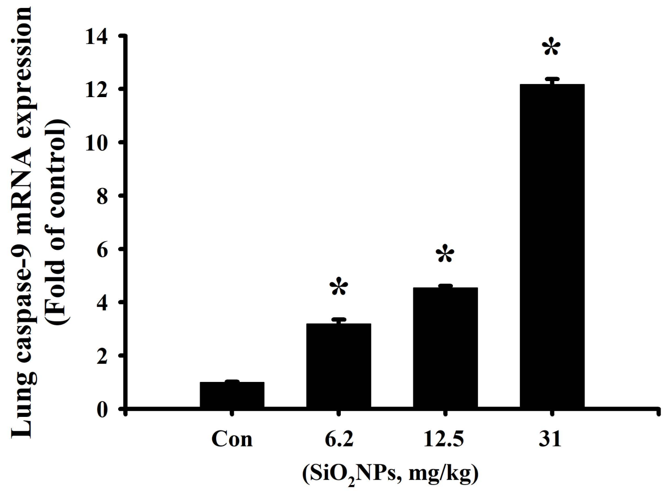

Supplementary Figure 6

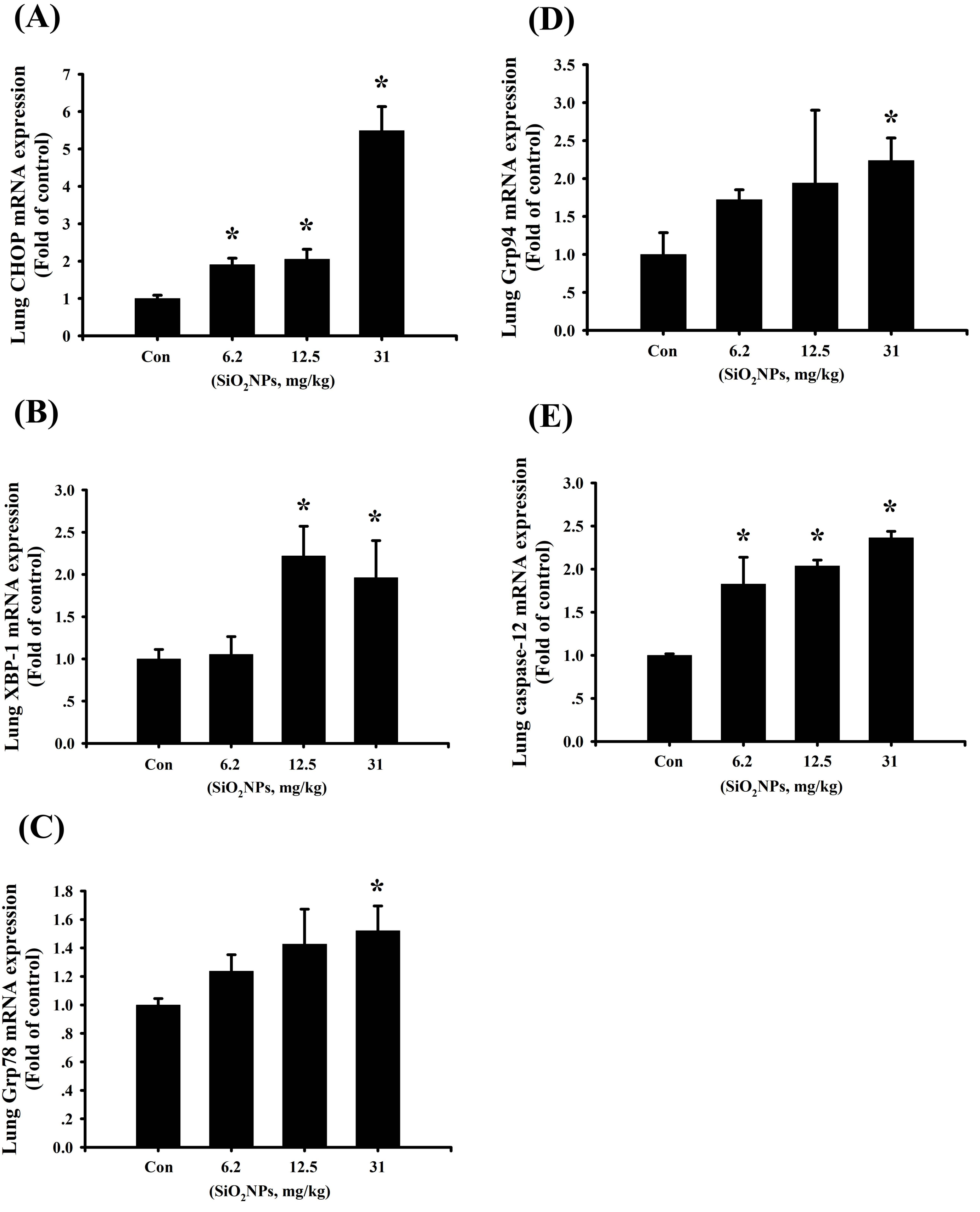

The corresponding full-length originals of Western blot analysis

Full-length originals of western blot image for Figure 2C

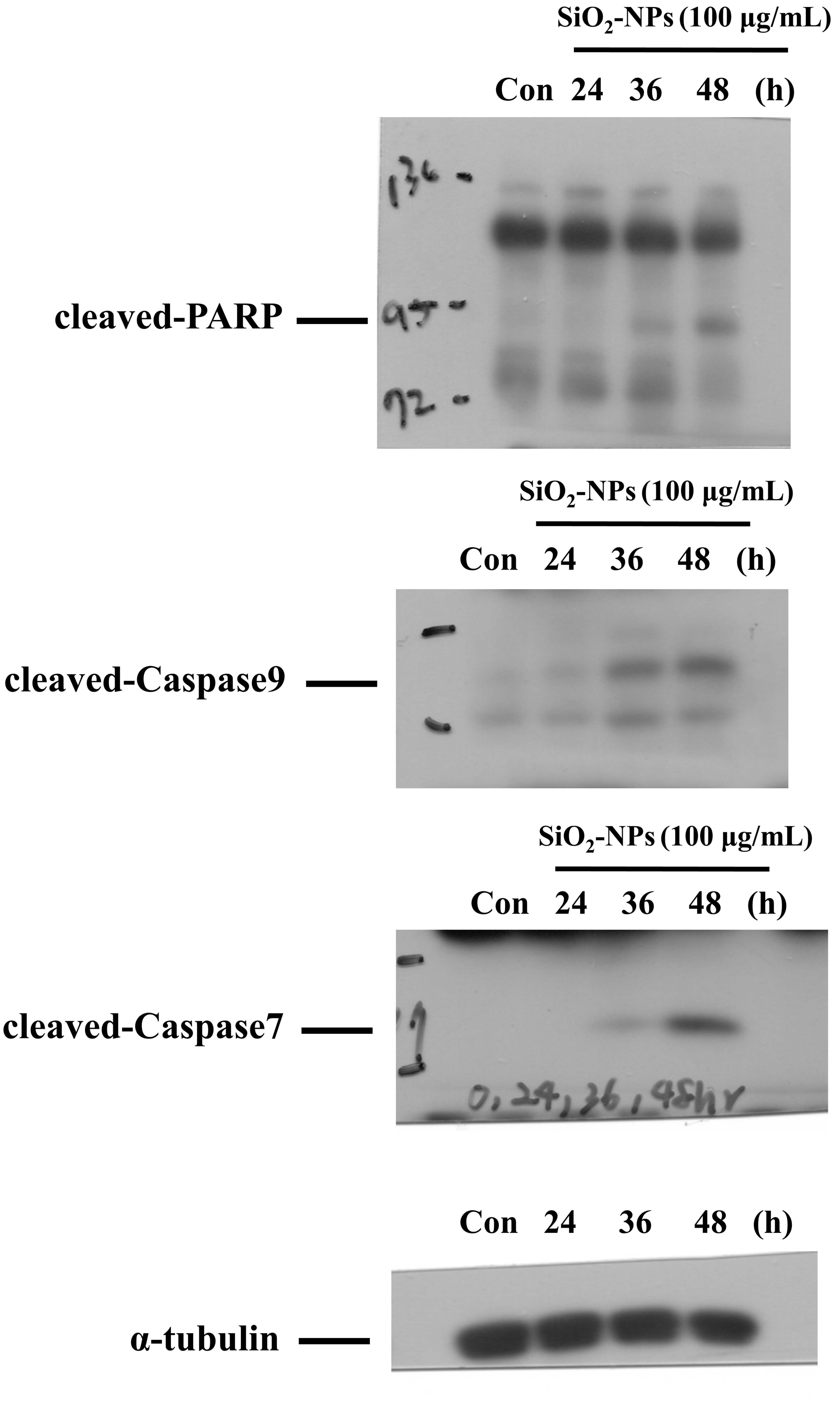

Full-length originals of western blot image for Figure 3C

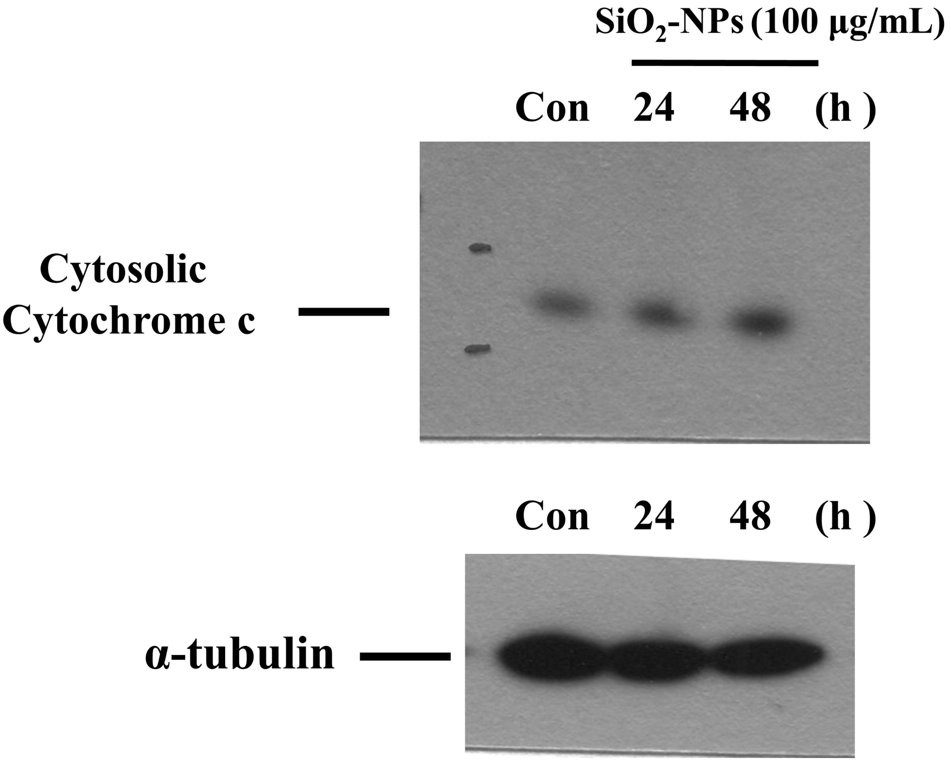

Full-length originals of western blot image for Figure 3D

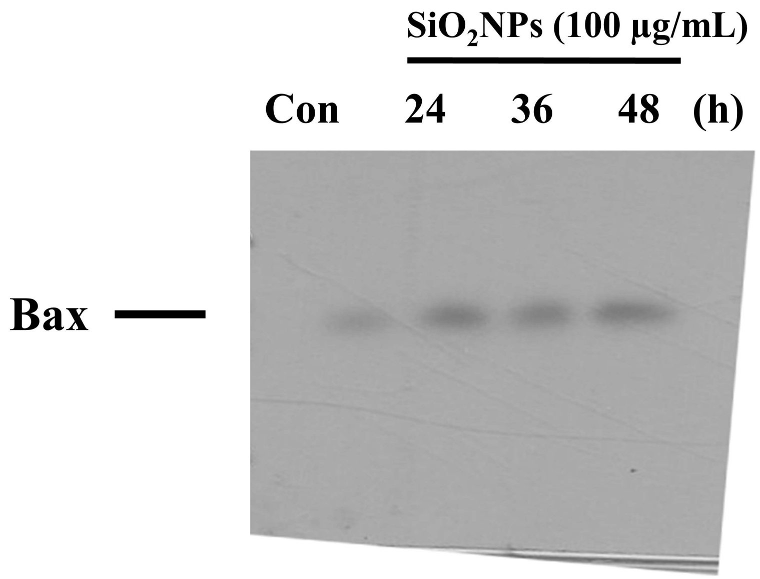

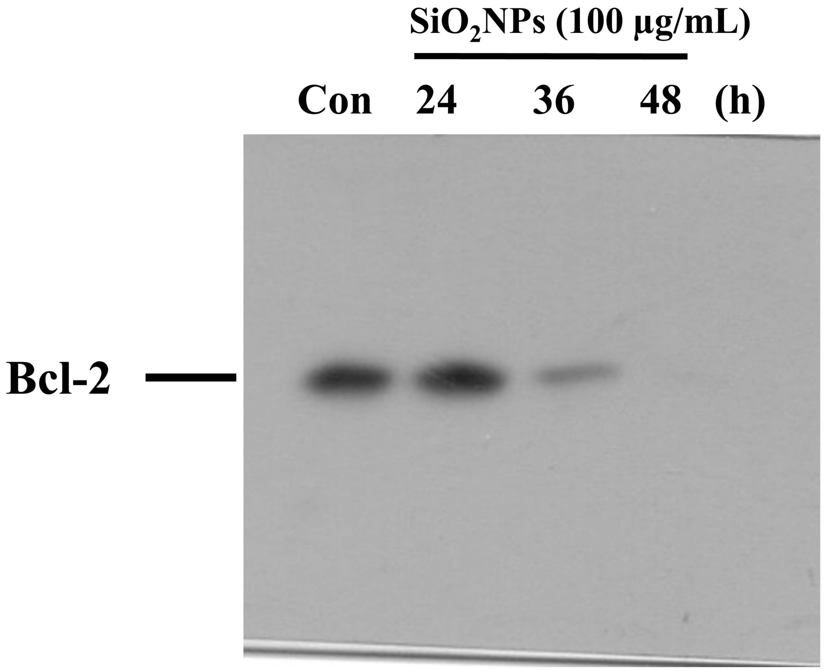

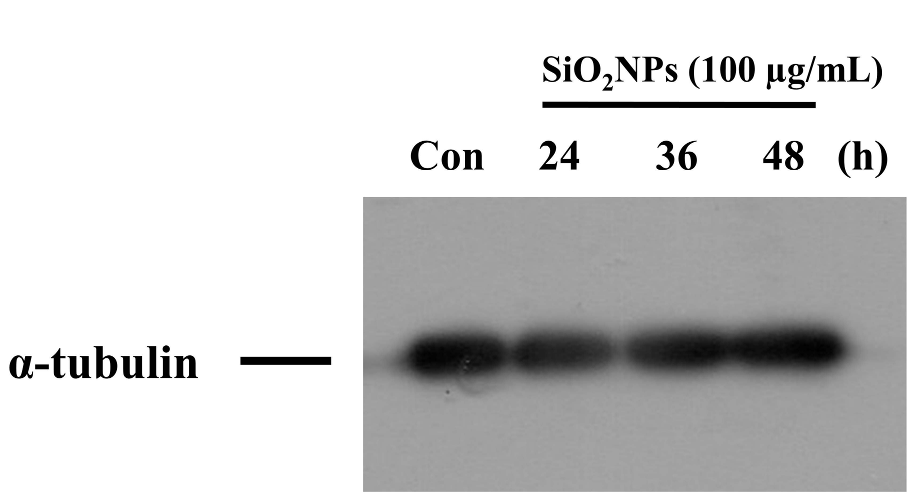

Full-length originals of western blot image for Figure 3F

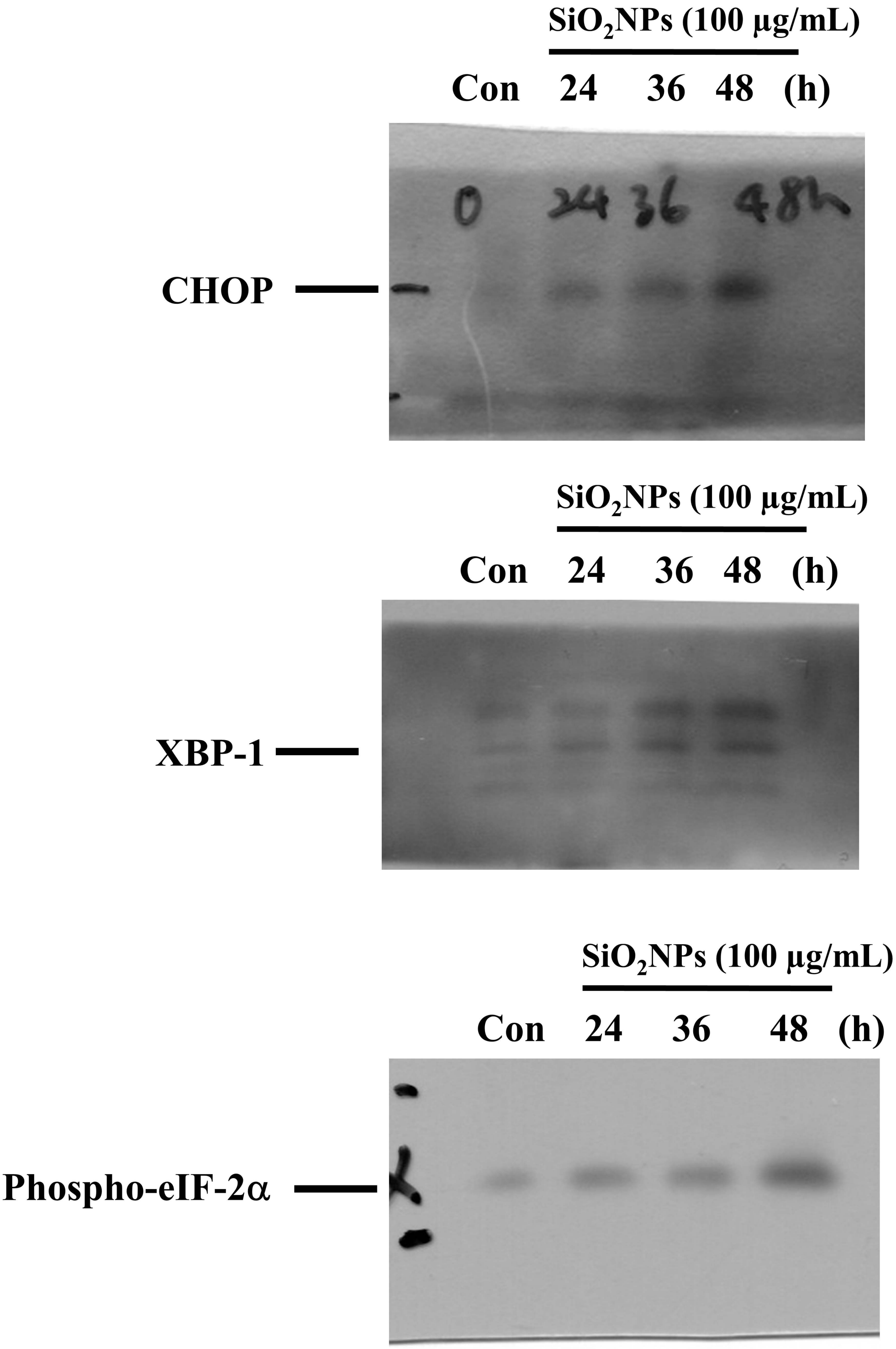

Full-length originals of western blot image for Figure 3F

SiO_2NPs (100 µg/mL)

Con 24 36 48 (h)

eIF-2α

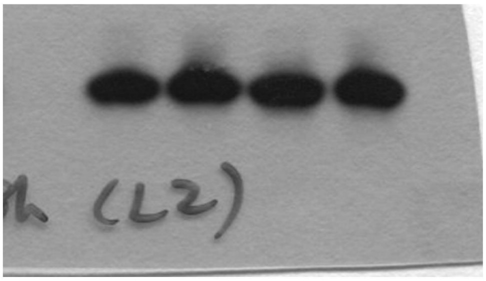

SiO_2NPs (100 µg/mL)

Con 24 36 48 (h)

Pro-Caspase 12

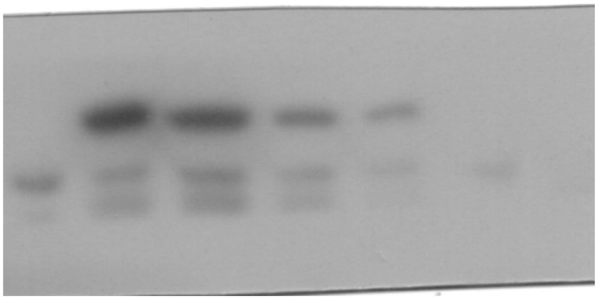

SiO_2NPs (100 µg/mL)

Con 24 36 48 (h)

α-tubulin

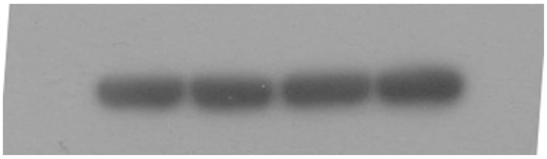

Full-length originals of western blot image for Figure 4B

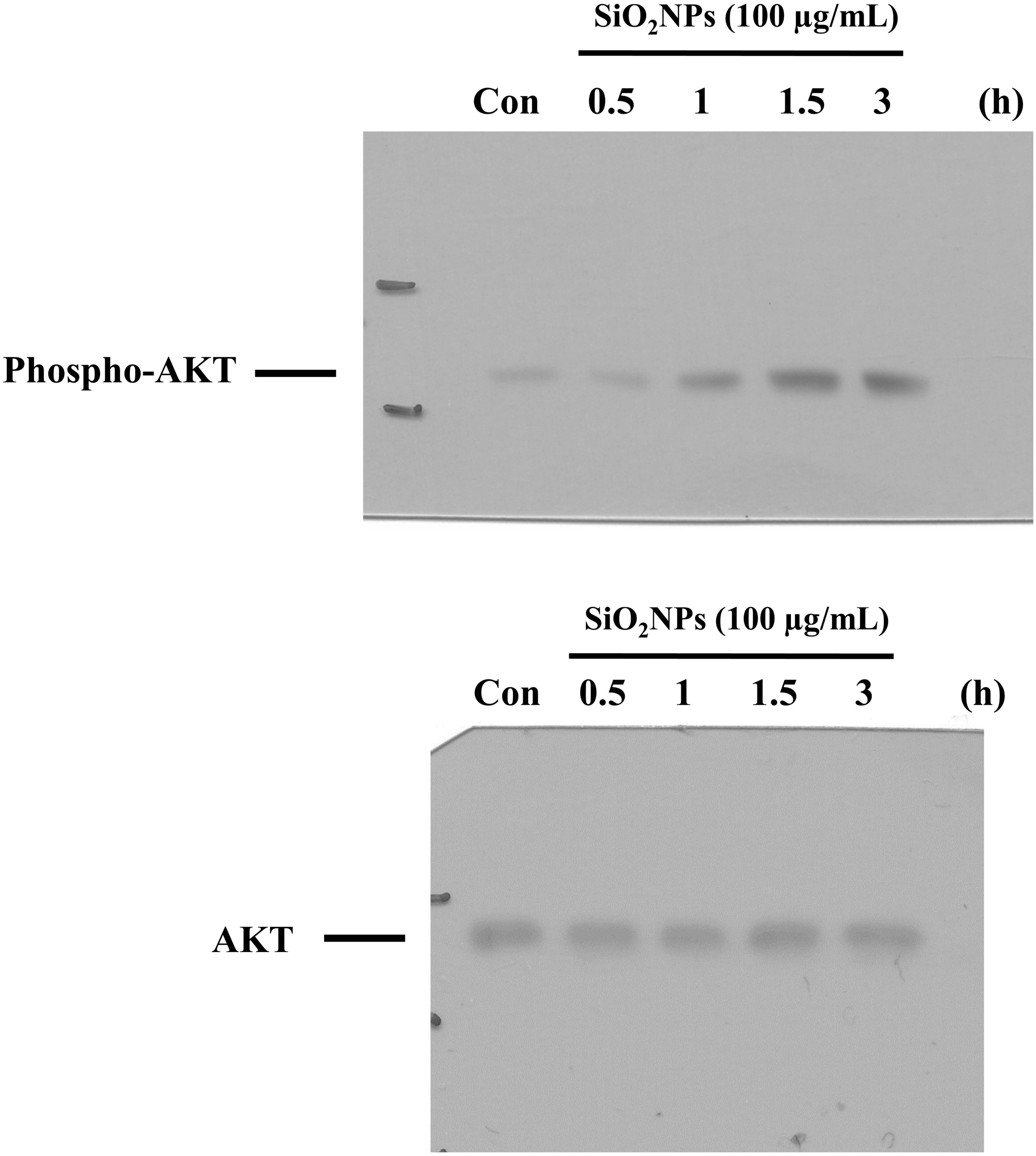

Full-length originals of western blot image for Figure 6B

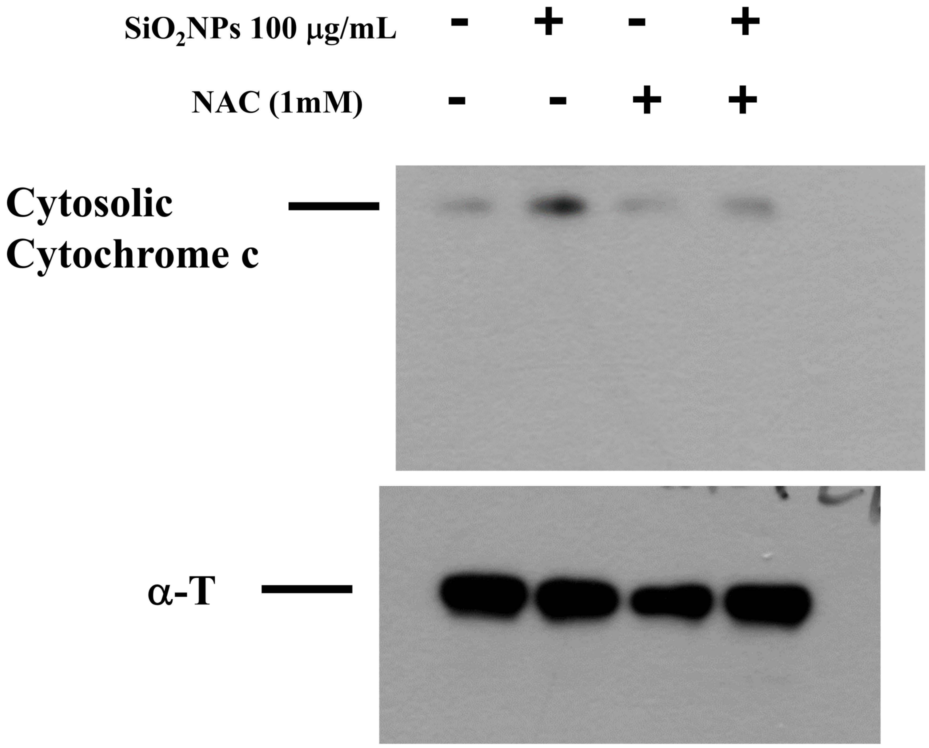

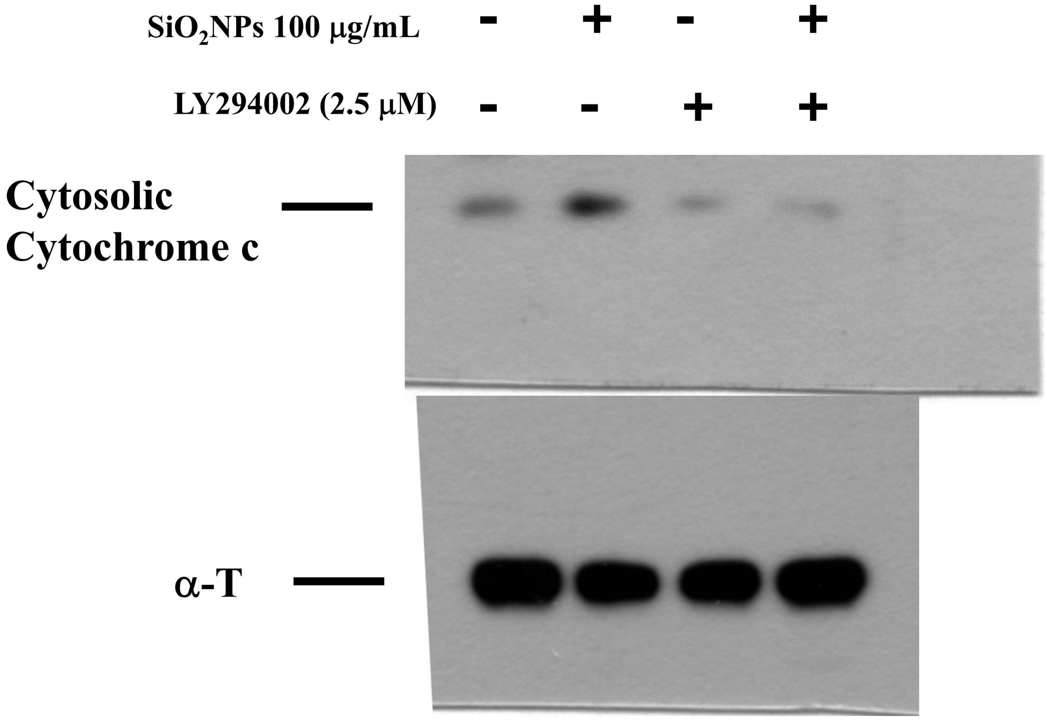

Full-length originals of western blot image for Figure 7A

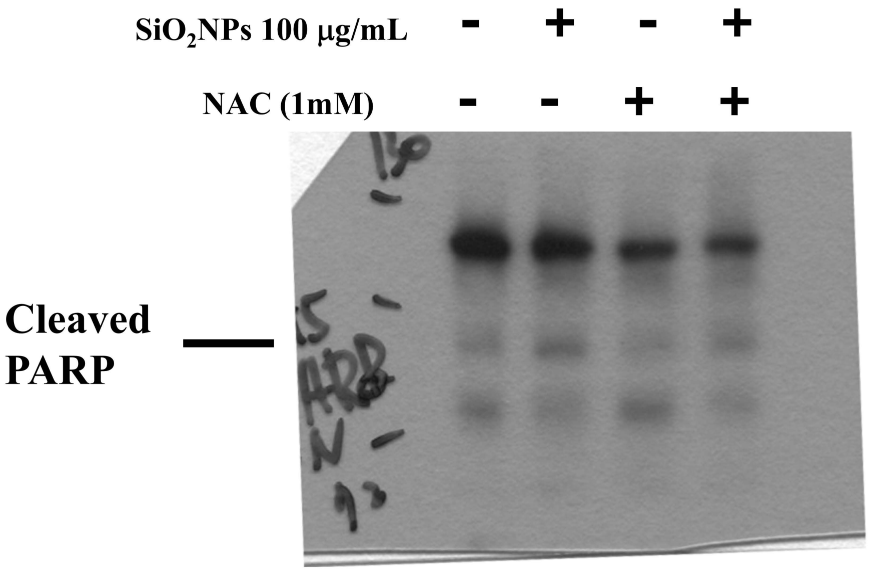

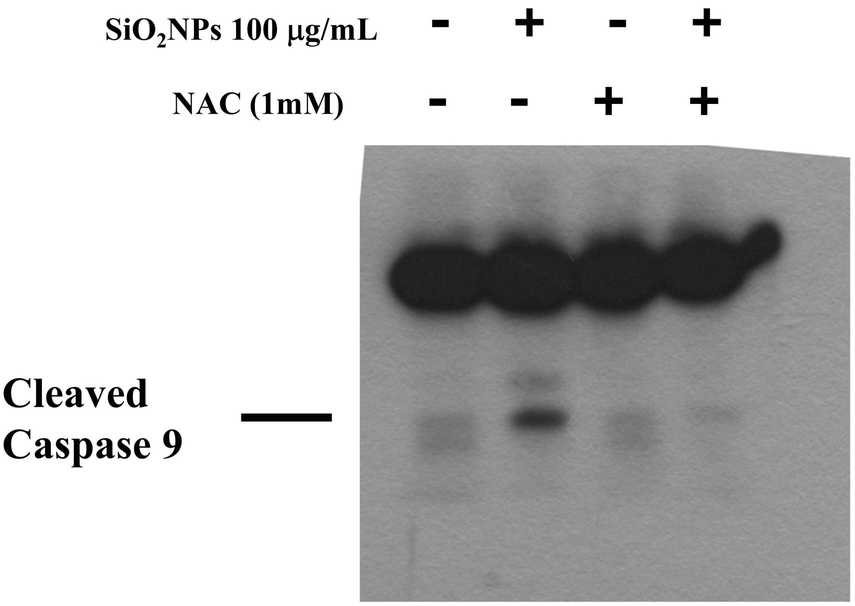

Full-length originals of western blot image for Figure 7A

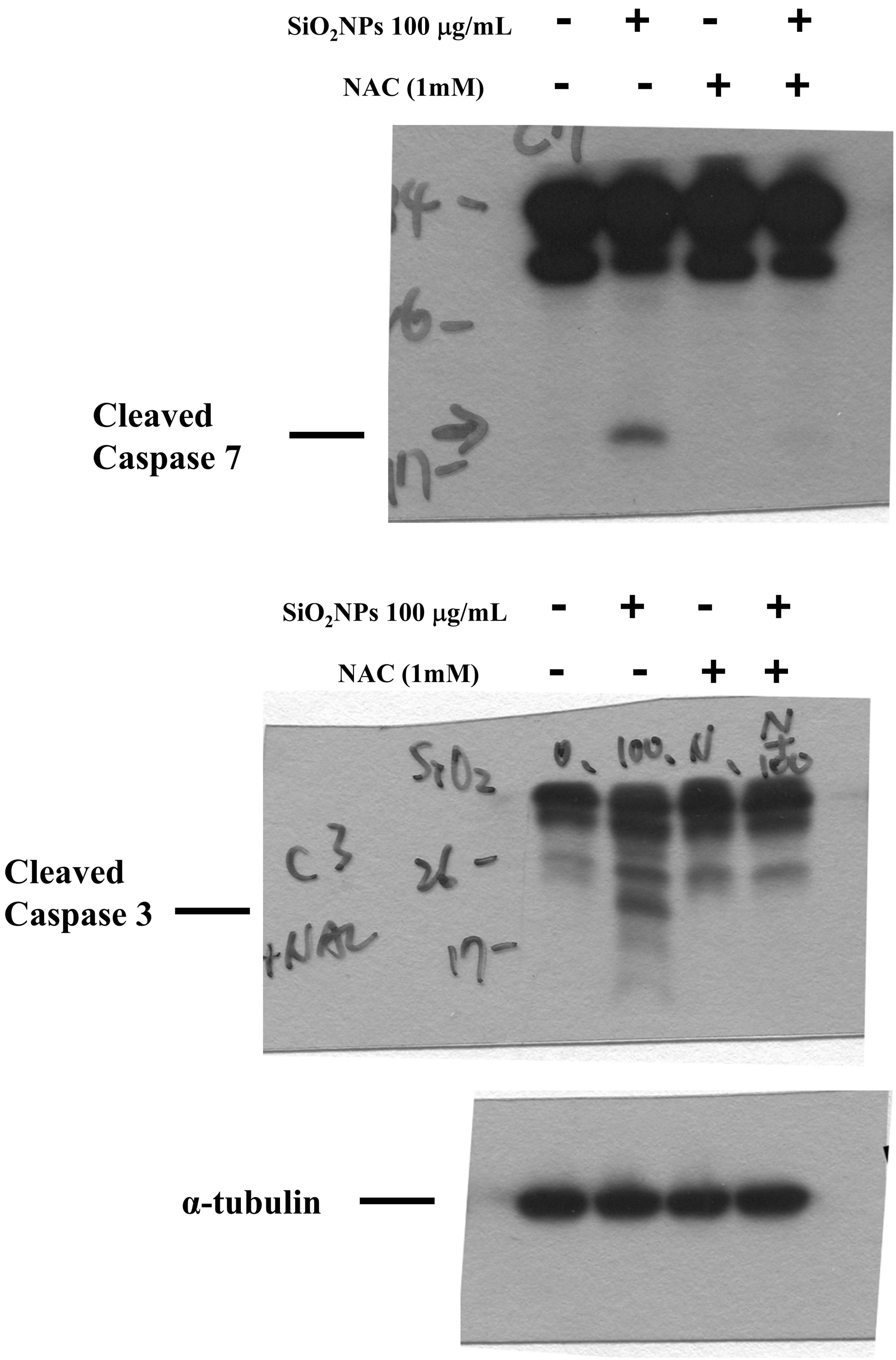

Full-length originals of western blot image for Figure 7B

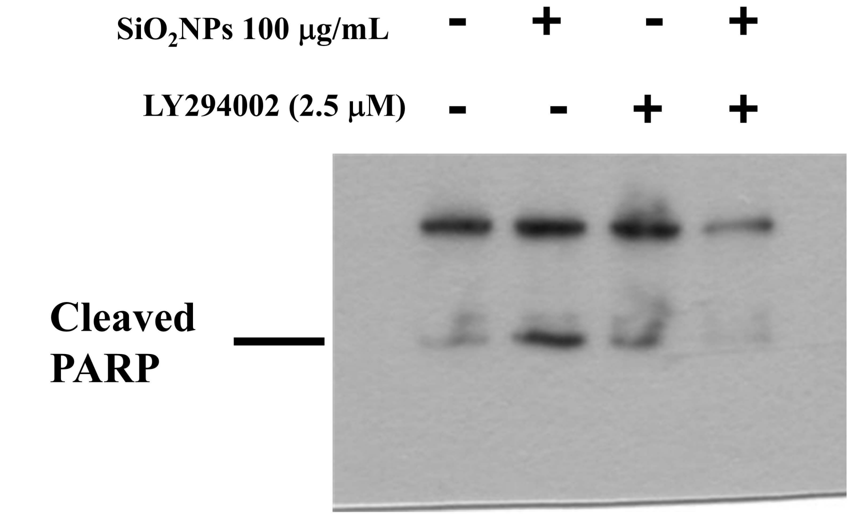

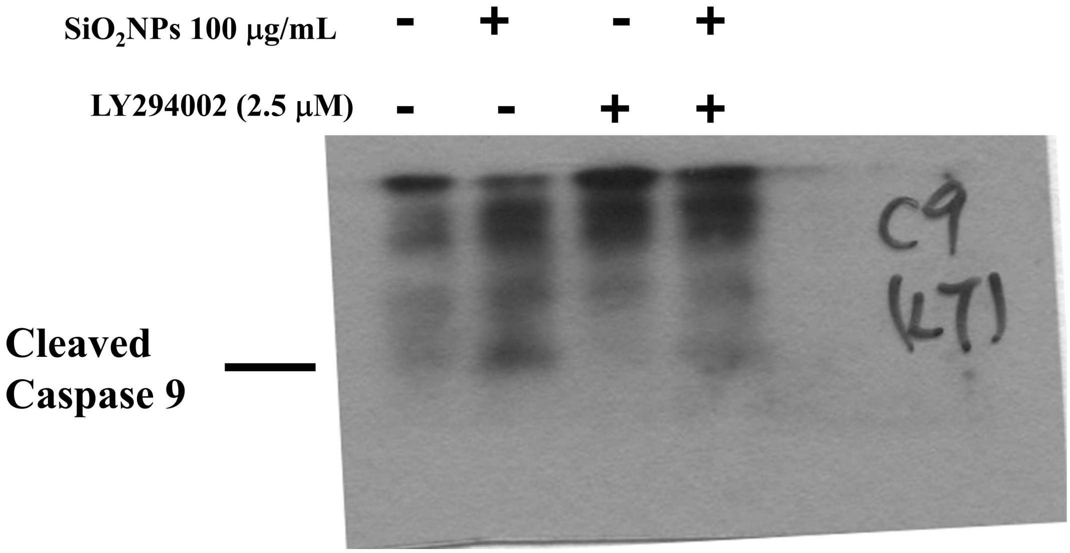

Full-length originals of western blot image for Figure 7B

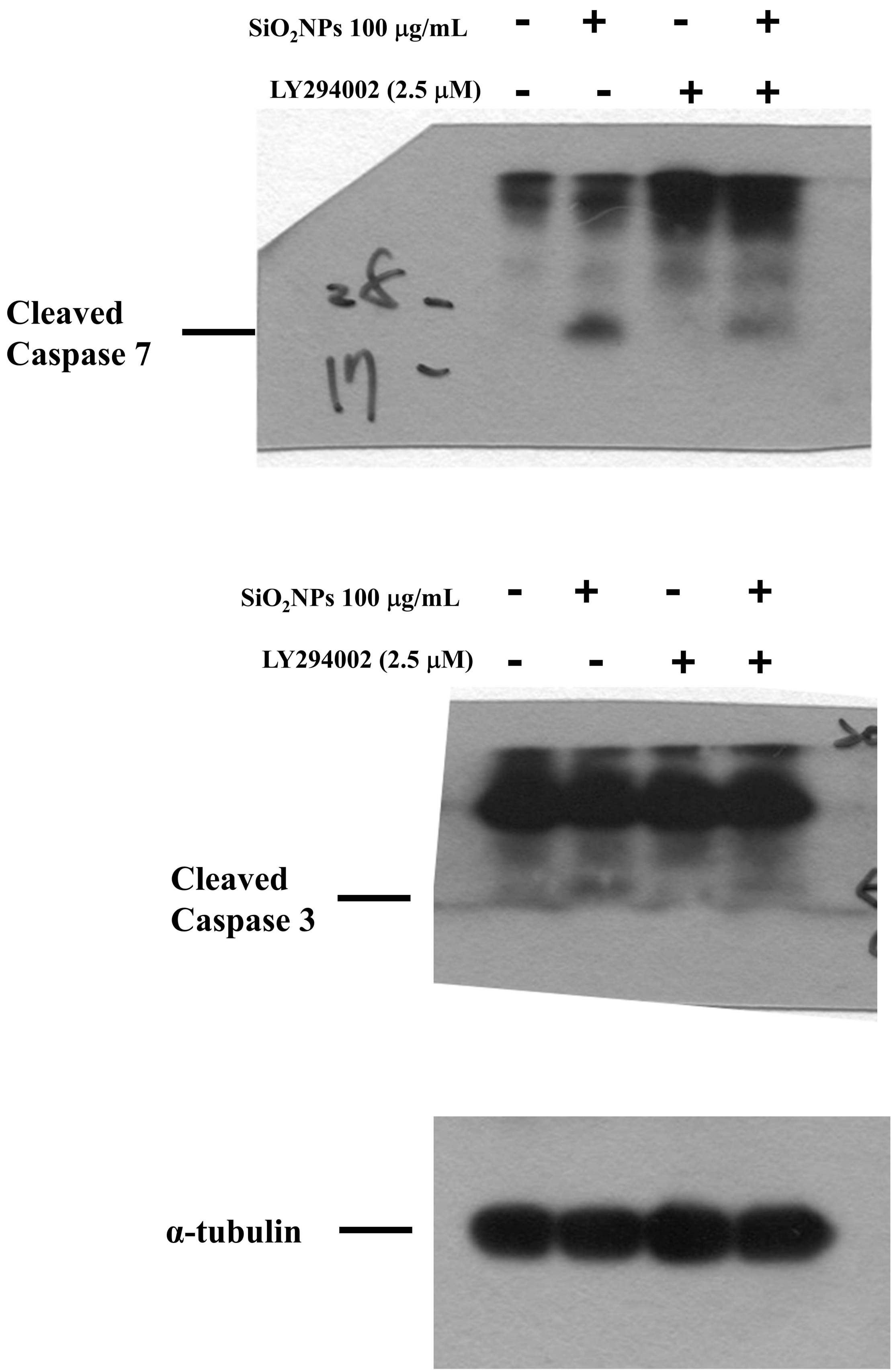

Full-length originals of western blot image for Figure 7C

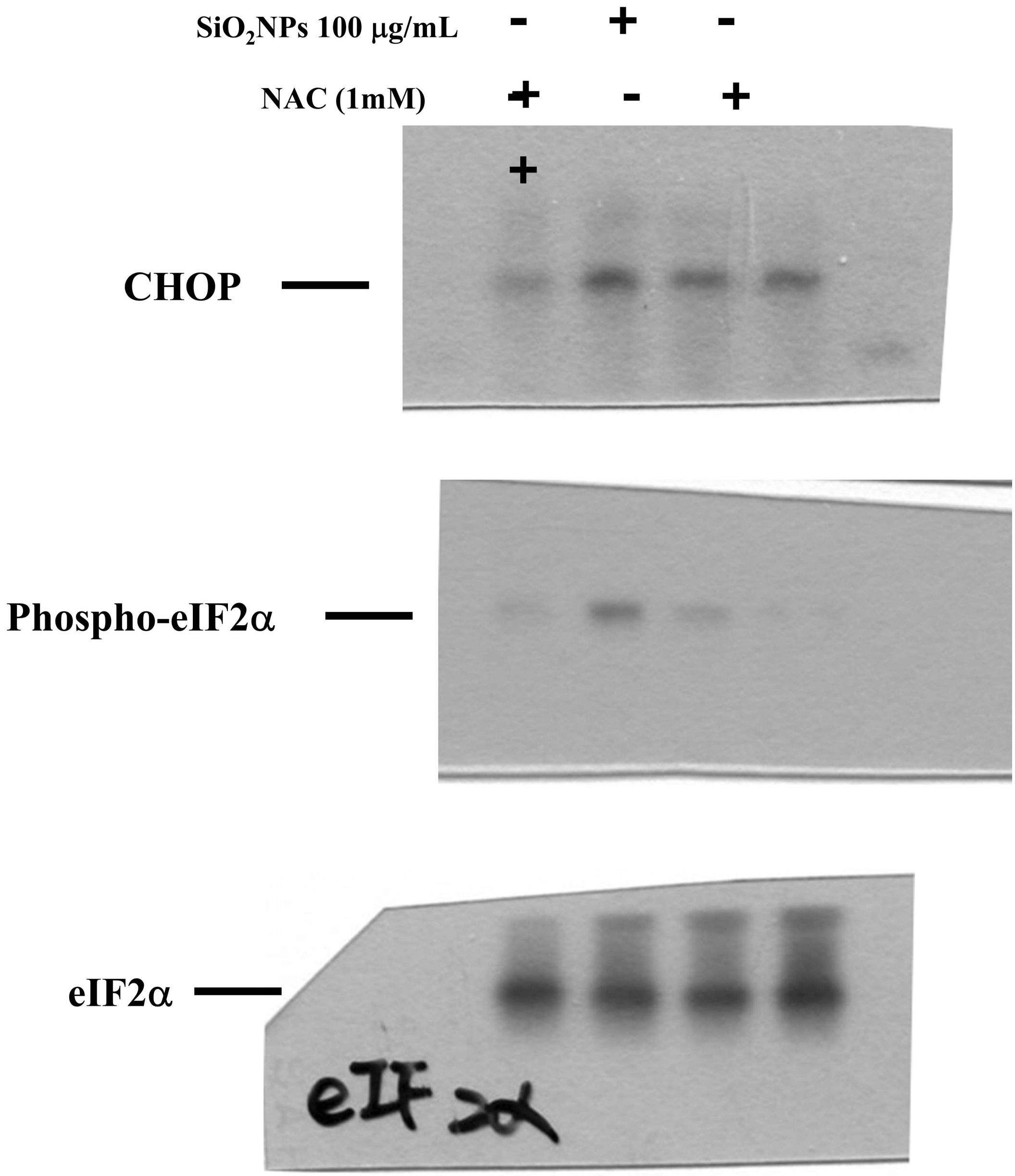

Full-length originals of western blot image for Figure 7C

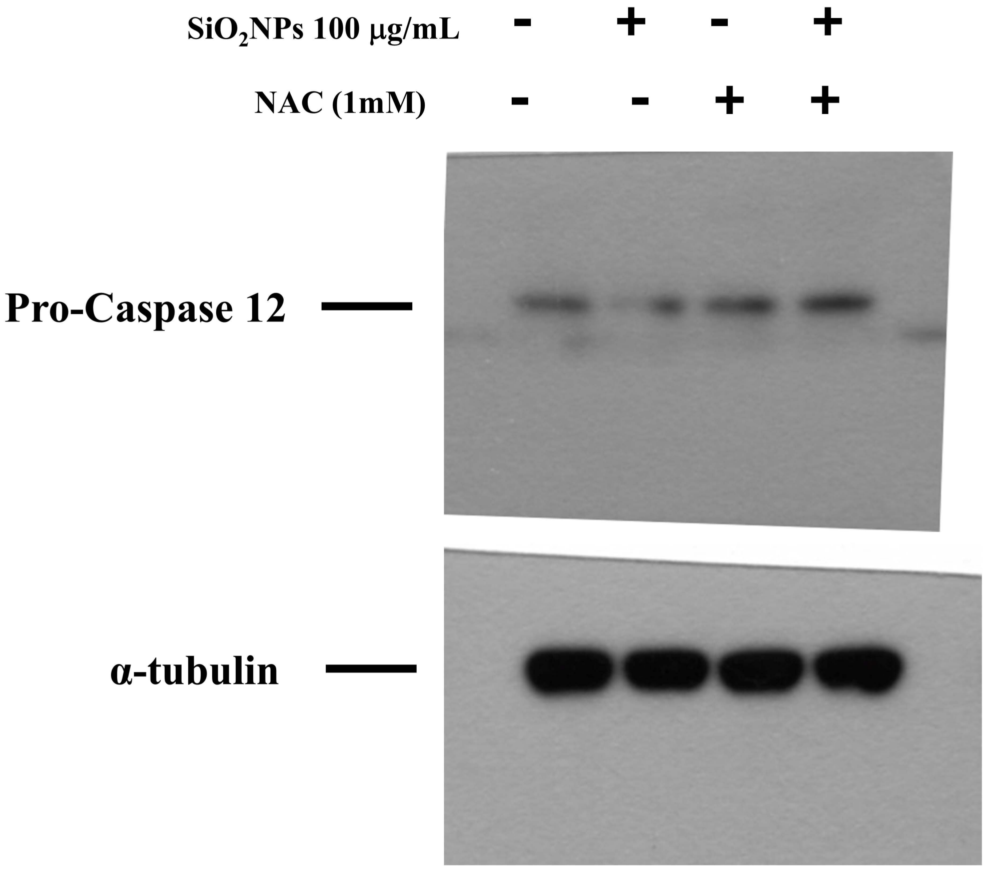

Full-length originals of western blot image for Figure 7D

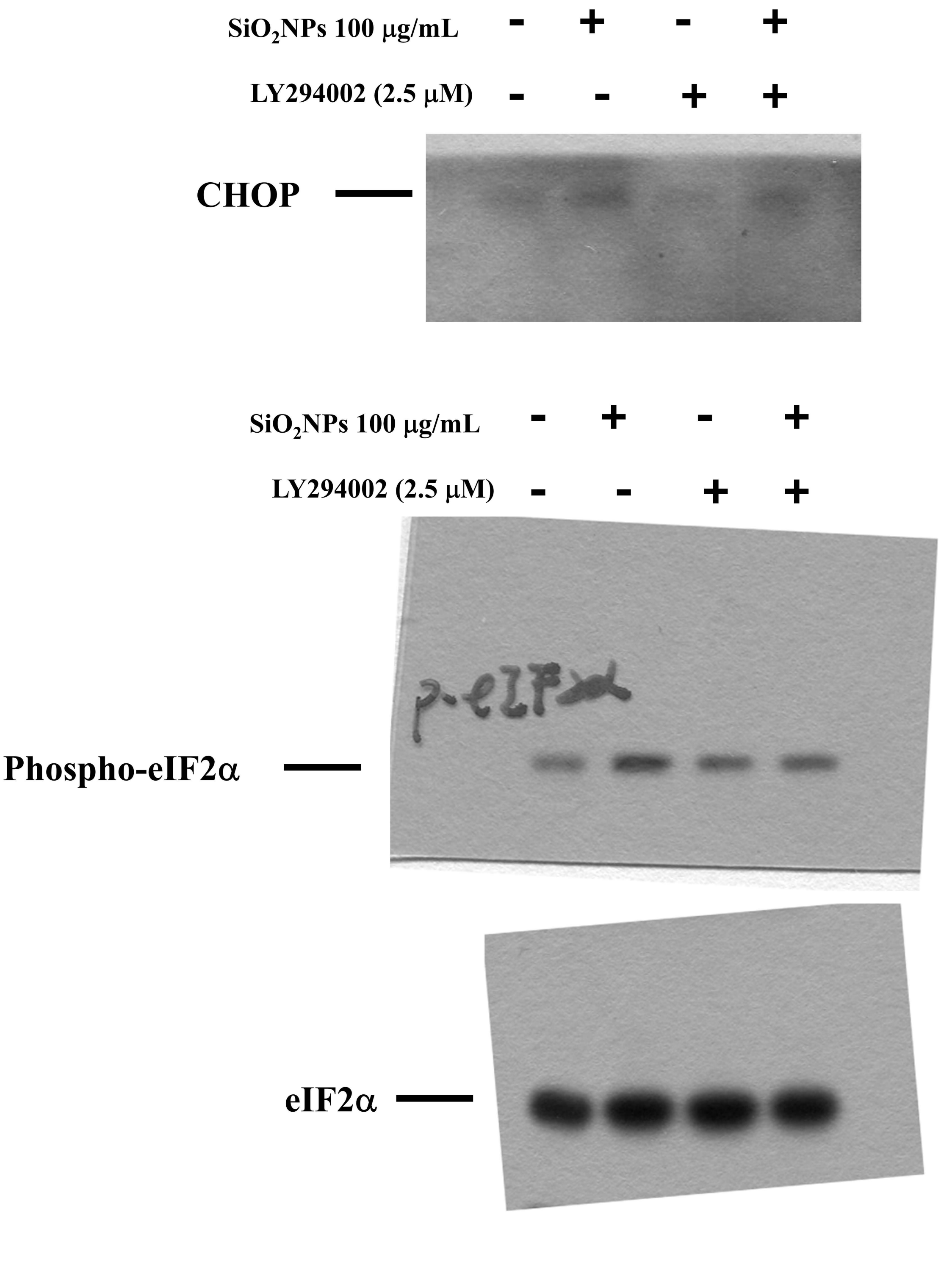

Full-length originals of western blot image for Figure 7D

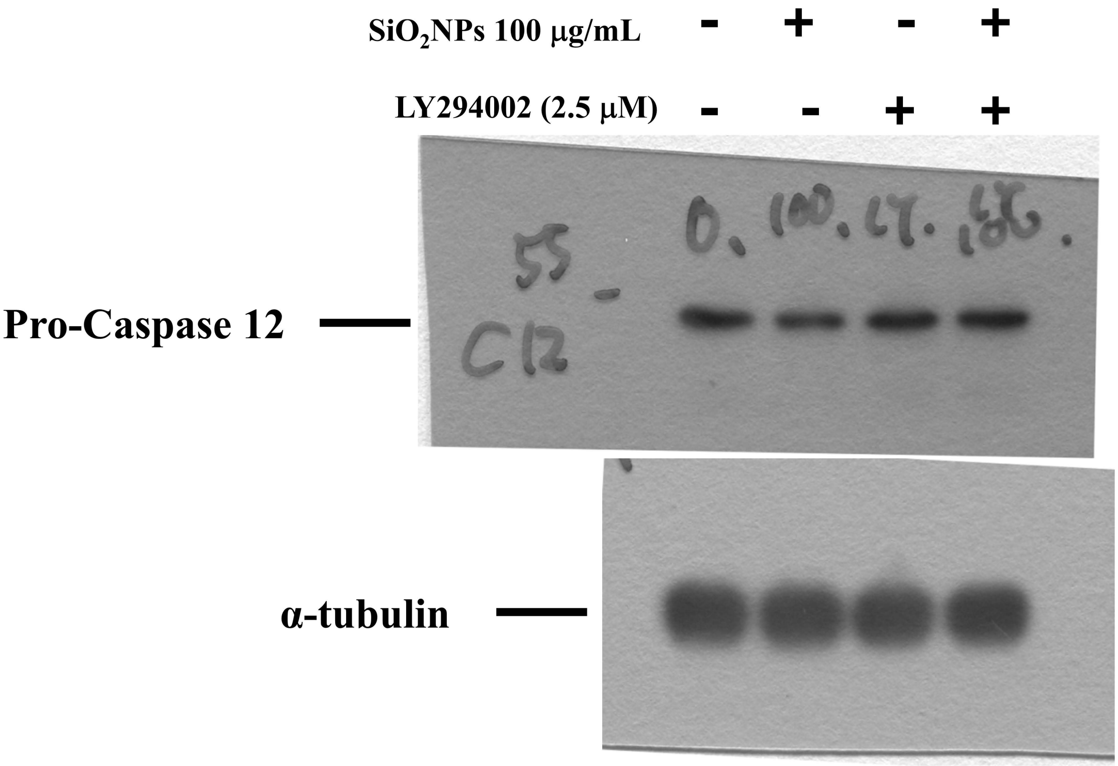

Full-length originals of western blot image for Figure 8C

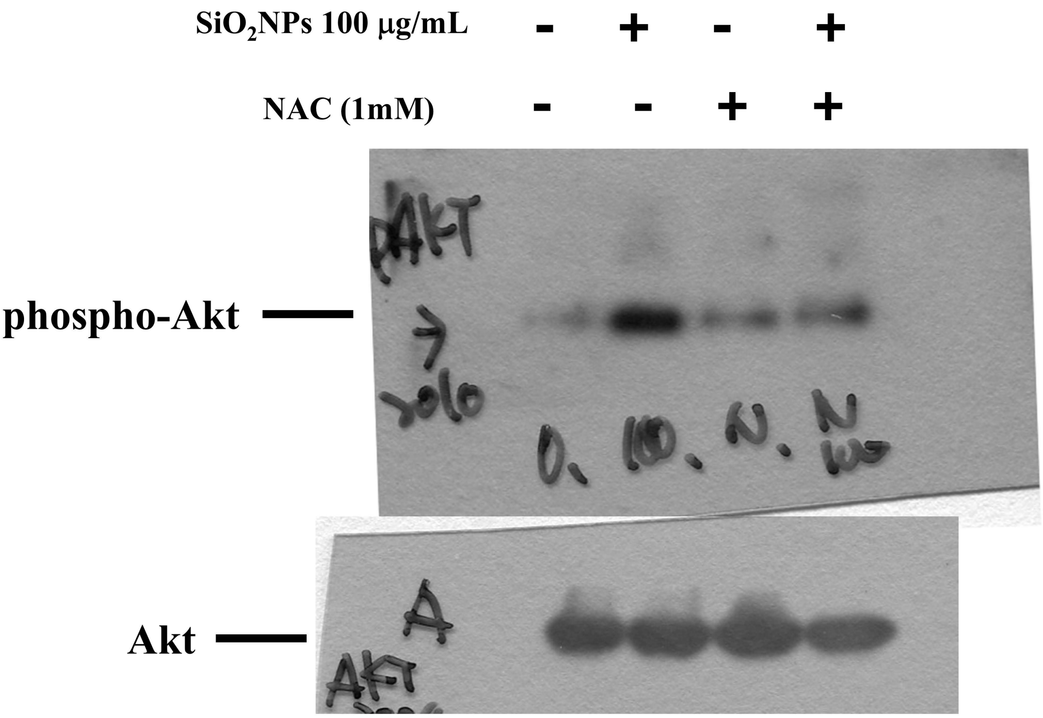

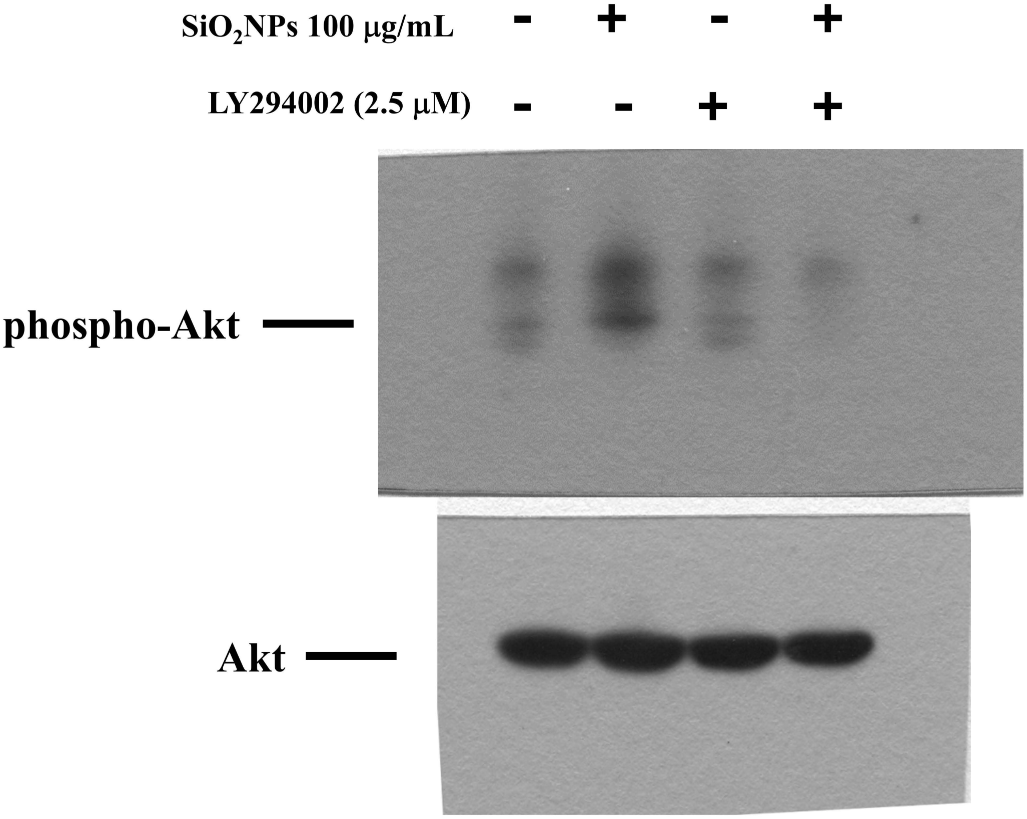

Full-length originals of western blot image for Supplementary Figure 2B

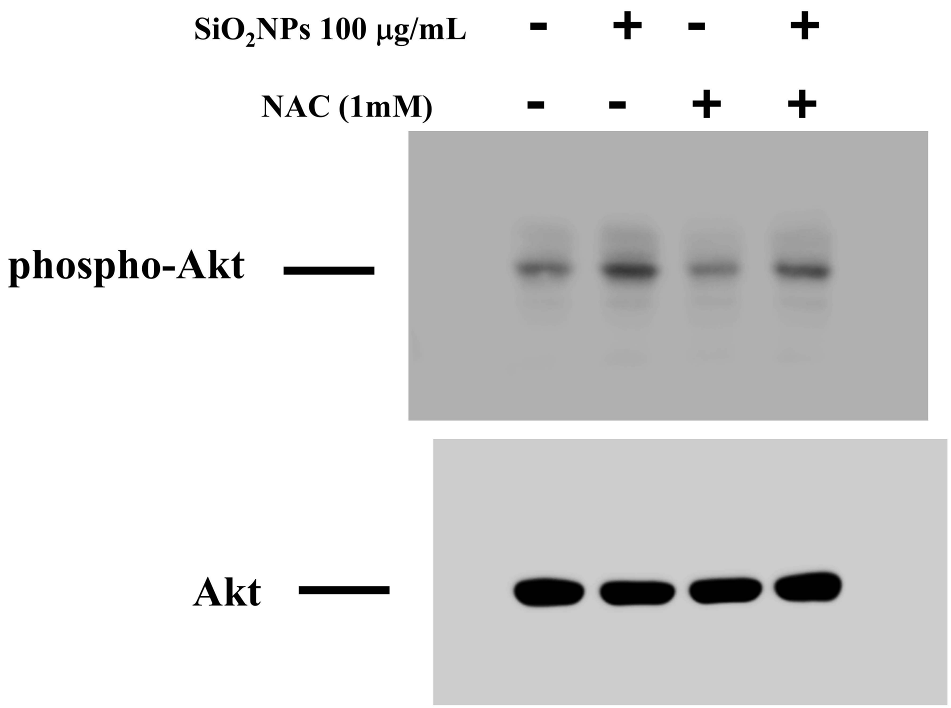

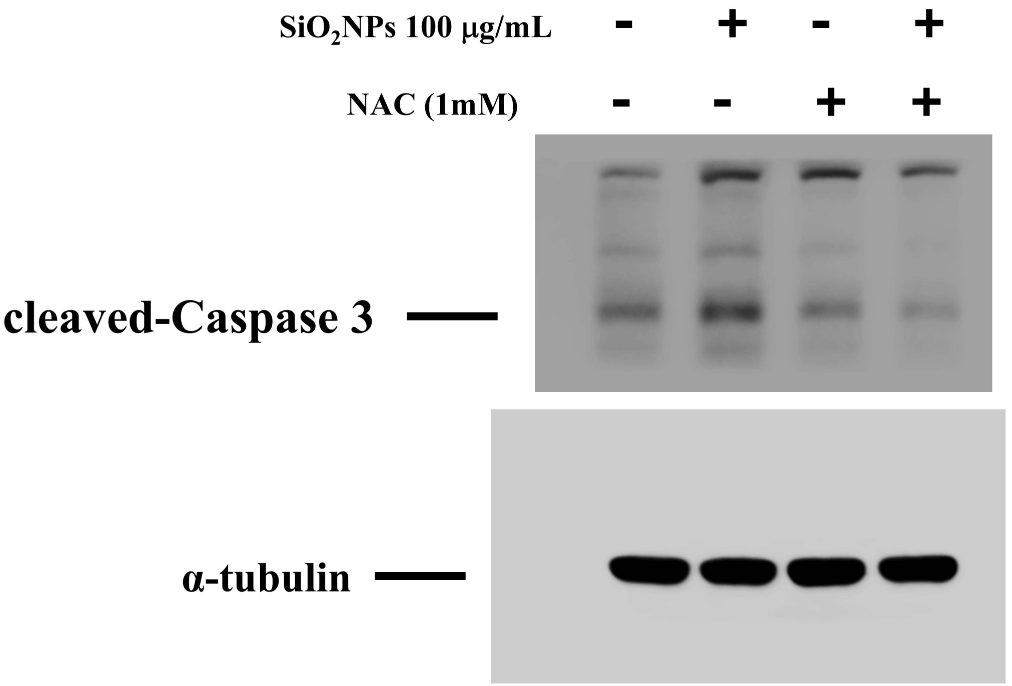

Full-length originals of western blot image for Supplementary Figure 2B

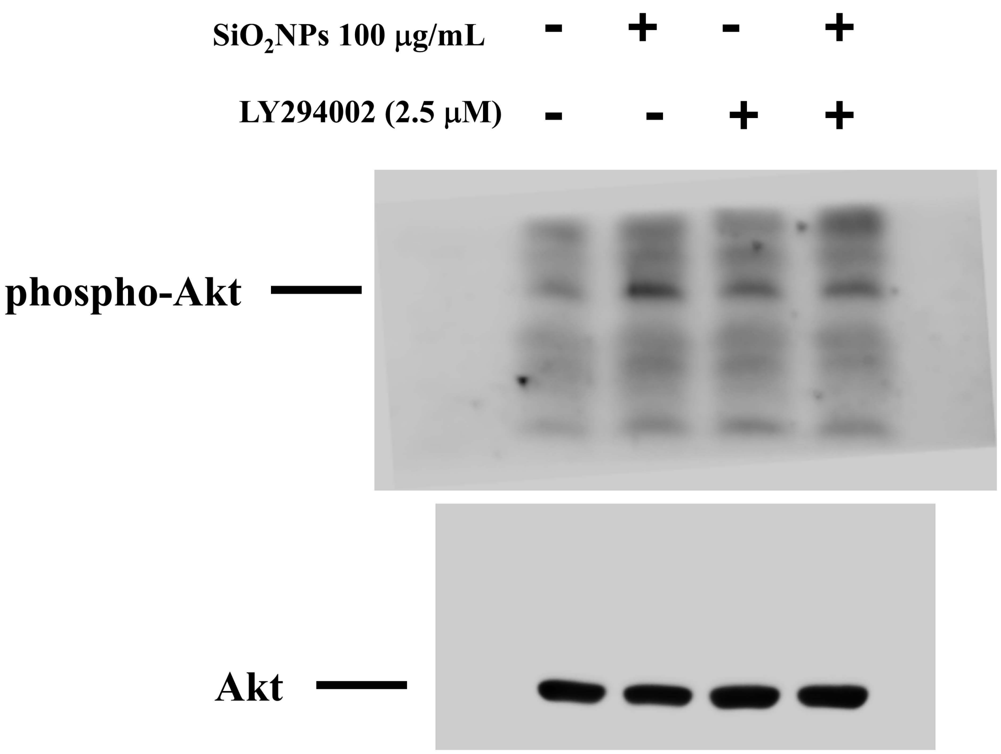

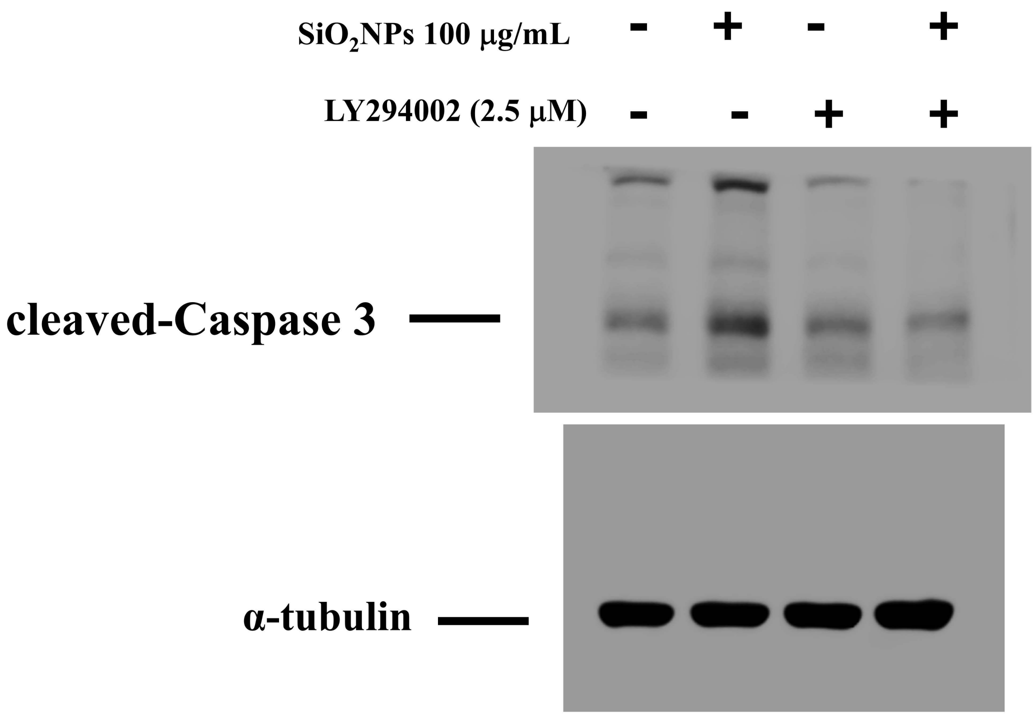

Supplement: Supplementary file 1 — Supplementary Information. [file 41598_2020_66644_MOESM1_ESM.pdf]
